# Influence of social deprivation on morbidity and all-cause mortality of cardiometabolic multi-morbidity: a cohort analysis of the UK Biobank cohort

**DOI:** 10.1186/s12889-023-17008-5

**Published:** 2023-11-07

**Authors:** Zhou Jiang, Shuo Zhang, Ping Zeng, Ting Wang

**Affiliations:** 1 Department of Biostatistics, School of Public Health, Xuzhou Medical University, Xuzhou, 221004 Jiangsu China; 2 Center for Medical Statistics and Data Analysis, Xuzhou Medical University, Xuzhou, 221004 Jiangsu China; 3 Key Laboratory of Human Genetics and Environmental Medicine, Xuzhou Medical University, Xuzhou, 221004 Jiangsu China; 4 Key Laboratory of Environment and Health, Xuzhou Medical University, Xuzhou, 221004 Jiangsu China; 5 Engineering Research Innovation Center of Biological Data Mining and Healthcare Transformation, Xuzhou Medical University, Xuzhou, 221004 Jiangsu China

**Keywords:** Townsend deprivation index, Cardiometabolic disease, Cardiometabolic multimorbidity, All-cause mortality, UK Biobank

## Abstract

**Background:**

The relation of social deprivation with single cardiometabolic disease (CMD) was widely investigated, whereas the association with cardiometabolic multi-morbidity (CMM), defined as experiencing more than two CMDs during the lifetime, is poorly understood.

**Methods:**

We analyzed 345,417 UK Biobank participants without any CMDs at recruitment to study the relation between social deprivation and four CMDs including type II diabetes (T2D), coronary artery disease (CAD), stroke and hypertension. Social deprivation was measured by Townsend deprivation index (TDI), and CMM was defined as occurrence of two or more of the above four diseases. Multivariable Cox models were performed to estimate hazard ratios (HRs) per one standard deviation (SD) change and in quartile (Q1-Q4, with Q1 as reference), as well as 95% confidence intervals (95% CIs).

**Results:**

During the follow up, 68,338 participants developed at least one CMD (median follow up of 13.2 years), 16,225 further developed CMM (median follow up of 13.4 years), and 18,876 ultimately died from all causes (median follow up of 13.4 years). Compared to Q1 of TDI (lowest deprivation), the multivariable adjusted HR (95%CIs) of Q4 (highest deprivation) among participants free of any CMDs was 1.23 (1.20 ~ 1.26) for developing one CMD, 1.42 (1.35 ~ 1.48) for developing CMM, and 1.34 (1.27 ~ 1.41) for all-cause mortality. Among participants with one CMD, the adjusted HR (95%CIs) of Q4 was 1.30 (1.27 ~ 1.33) for developing CMM and 1.34 (1.27 ~ 1.41) for all-cause mortality, with HR (95%CIs) = 1.11 (1.06 ~ 1.16) for T2D patients, 1.07 (1.03 ~ 1.11) for CAD patients, 1.07 (1.00 ~ 1.15) for stroke patients, and 1.24 (1.21 ~ 1.28) for hypertension patients. Among participants with CMM, TDI was also related to the risk of all-cause mortality (HR of Q4 = 1.35, 95%CIs 1.28 ~ 1.43).

**Conclusions:**

We revealed that people living with high deprived conditions would suffer from higher hazard of CMD, CMM and all-cause mortality.

**Supplementary Information:**

The online version contains supplementary material available at 10.1186/s12889-023-17008-5.

## Introduction

Cardiometabolic diseases (CMDs) are the leading cause of premature mortality worldwide and produce extremely high socioeconomic burden on healthcare resources [1]. We here focus on four CMDs including type II diabetes (T2D), coronary artery disease (CAD), stroke, and hypertension because they are more likely to co-occur [2]. Further, these diseases have been widely analyzed simultaneously in previous studies [3, 4]. One may experience one or more of these diseases during the lifetime, which is defined as cardiometabolic multi-morbidity (CMM) [2–4]. With the rise in population aging and the advancements in medical healthcare, over the past few decades there has been a rapid increase in the number of people who suffered from CMM [5–7]. Furthermore, a history of CMM was associated with 12–15 years reduction of life expectancy at the age of 60 years [8]; thus, a special attention should be paid on the adverse consequence of CMM.

Although it has never stopped finding out the biological and clinical mechanism of CMDs, there is a growing awareness that socioeconomic factors also play an important role in the course of these diseases and that more considerate and broad prevention strategies are urgently needed [9, 10]. Among those, socioeconomic deprivation is a commonly-used aggregative measurement of socioeconomic statuses including unemployment, car and home ownership, and household overcrowding, presenting an advantage in quantifying health inequality [11, 12]. People with high social deprivation typically have higher burden of adverse lifestyle behaviors such as cigarette smoking, psychosocial stress, and limited access to health conditions, which in turn cause increased risk of CMDs [9, 13].

The association between high socioeconomic deprivation and increased risk of new-onset CMD and mortality has been well documented in literature. For example, elevated socioeconomic deprivation was shown to associate with greater risk of T2D, hospitalization, and related mortality [14–19]. A cohort study conducted in 7-county region in US reported that area deprivation was linked with higher risk of 18 chronic condition including stroke, diabetes and CAD [20]. Consistently, the adverse impacts of social deprivation on individual CMDs have already been discovered in other studies [21–30]. Particularly, recent studies in the UK Biobank showed Townsend deprivation index (TDI) was related to higher CAD and mortality risk [31, 32].

Understanding the intricate association between social deprivation and the whole course from CMD to CMM or to mortality can provide insightful evidence linking social inequality and CMM/death progression trajectory. To our knowledge, the relation between social deprivation and CMM and CMM-related mortality has not been completely explored thus far. To fill this gap, in the present work we aim to perform a comprehensive exploration to assess whether and to what extent social deprivation is associated with CMD, CMM, and mortality under different baseline conditions using data source of over 0.5 million UK Biobank participants [33]. Furthermore, subgroup and sensitivity analyses were performed to evaluate robustness and potential variation of our findings; attributable risk percent (AR%) and population attributable risk percent (PAR%) of TDI was also calculated.

## Methods

### Study population

**Fig. 1:**
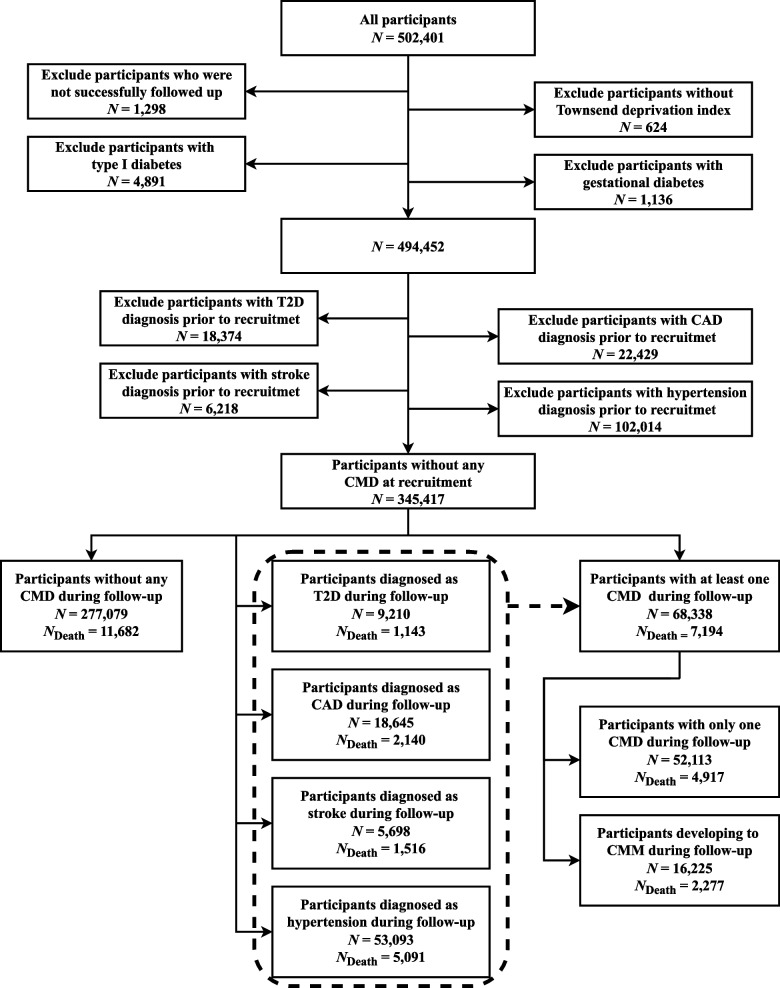
Flowchart of participant enrollment. T2D, type II diabetes; CAD, coronary artery disease; CMD, Cardiometabolic disease; CMM, cardiometabolic multi-morbidity defined as the condition of suffering from at least two of the four studied diseases. *N*_Death_: number of deaths

The UK Biobank cohort recruited over half a million community-dwelling individuals aged 37–73 years from 22 dedicated centers across the UK between 2006 and 2010, and conducted a comprehensive touchscreen assessment through questionnaires, physical measurements, and biological sample testing [33]. In our study, participants who met any of the following criteria at baseline were excluded: (1) withdrawn from the survey (*N* = 1,298), (2) had missing data in TDI (*N* = 624), (3) suffered from type I diabetes (*N* = 4,819) or gestational diabetes (*N* = 1,136), and (4) suffered from any of CMDs (*N* = 18,374 for T2D, *N* = 22,429 for CAD, *N* = 6,218 for stroke, and *N* = 102,014 for hypertension) before recruitment. Finally, 345,417 “healthy” individuals without any CMD at recruitment were included in our subsequent analyses (Fig. 1).

### Variable selection and definition

#### Social deprivation

Social deprivation was quantitatively measured by TDI (Field 189) which derived from the postcode of residence by aggregating data of unemployment, car and home ownership, and household overcrowding [12].

#### Morbidity and mortality of cardiometabolic diseases

The four CMDs were mainly ascertained via ICD-10 codes (Supplemental File: Table S1). Some other records were also used, such as self-reported illness (Field 20002) for all the four diseases, medical conditions (Fields 6153 and 6177) for T2D and hypertension, doctor-diagnosed vascular/heart problems (Field 6150) for CAD, stroke, and hypertension. We also considered all stroke cases (i.e., ischemic stroke, intracerebral hemorrhage, and subarachnoid hemorrhage) that were algorithmically determined through cross-checking over multiple data sources [34].

Disease-specific diagnosis date was retrieved from linked hospital records including Health Episode Statistics (England), the Scottish Morbidity Records (Scotland), and the Patient Episode Database (Wales). CMM was defined as the situation where a patient suffered from at least two types of the four diseases, and CMM onset was determined as the date when the second disease was diagnosed. All-cause mortality and death date were obtained through death certificates held by the National Health Service (NHS) Information Centre (England and Wales) and the NHS Central Register (Scotland).

In our UK Biobank cohort, the complete date of follow-up was 19 July 2022; we employed it as the censoring date or used it to calculate survival time (see details in the Section of Statistical analysis).

#### Covariates

Several risk covariates were taken into account, including age (Field 21022), sex (Field 31), ethnicity (Field 21000), education (Field 6138), income (Field 738), former smoking status (Fields 1249 and 20160), former drinking status (Fields 3731 and 20117), physical activity (Field 22032), healthy diet score (Fields 1289, 1299, 1309, 1319, 1329, 1339, 1349, 1369, 1379, and 1389), body mass index (BMI) (Field 21001), systolic blood pressure (SBP) (Fields 93 and 4080), and diastolic blood pressure (DBP) (Fields 94 and 4079). For covariates with multiple measurements, we calculated the average for continuous variables, and screened across all measurements to examine the presence for categorical variables, considering simultaneously the use of as much data as possible and time sequence of causality. For example, we calculated the average for four times assessment of BMI. In addition, if there had a former smoking history at any measurement for a given participant, we considered this participant to be a former smoker although she/he might be reported as a former non-smoking at the initial visit. For cases, only assessment visit before onset of disease was taken into account. More details regarding the definition and coding of these covariates are described in the Supplemental File.

### Statistical analysis

#### Survival time, exploratory analysis, and Cox model

Survival time of each participant was calculated as the duration from the baseline to the date of diagnosis, death, or censoring (19 July 2022), whichever came first. The restricted cubic spline curve [35] was drawn and monotonic exposure–response relationship was observed (Supplemental File: Figure S1). The cumulative risk curve was also created for the quartile of TDI, with the first quartile (lowest deprivation) as the reference (Q1 vs. Q2, Q3, or Q4) (Supplemental File: Figure S2).

The associations of TDI with morbidity and mortality of these diseases were evaluated by multivariable Cox proportional hazards (PH) model. For participants free of any CMDs at recruitment (*N* = 345,417), we examined the association of TDI with onset of single CMD and CMM and all-cause mortality; for those participants diagnosed as at least one CMD (*N* = 68,338), we estimated the association of TDI with CMM onset and all-cause mortality; for participants suffering from CMM (*N* = 16,225), we evaluated the association of TDI with all-cause mortality. The PH assumption was evaluated via the method given in [36] and no obvious violation of this assumption was detected (*P* > 0.05). The hazard ratio (HR) per one standard deviation (SD) change or in quartile and its 95% confidence intervals (CIs) were reported. All analyses were performed within the R software environment (version 4.1.3). The *P* value was two-sided, with the statistical significance threshold set to 0.05.

#### Covariate adjustment and missing value treatment

We controlled for age, sex, ethnicity, education, income, smoking status, drinking status, physical activity, healthy diet score, BMI, SBP, and DBP when analyzing T2D, CAD, stroke, but did not consider SBP and DBP when analyzing hypertension. Multivariate imputations with chained equations (MICE) was implemented to impute missing value of each covariate [37]. We generated ten imputed datasets and combined their estimates according to Rubin’s rule [38].

#### Subgroup and sensitivity analyses

Considering that the association pattern between TDI and CMD likely differed by distinct levels of covariates such as gender [22, 30] and ethnicity [13], we conducted several subgroup analyses to examine robustness and potential variation of our findings. Specifically, we repeated the analysis in sub-studies stratified by sex (male or female), age (< 60 or > = 60 years old, defined by WHO [39]), income (< £31,000 or ≥ £31,000) [40], education (with or without college/university degree) [41], smoking status (former smoking or non-smoking), drinking status (former drinking or non-drinking), physical activity (low, moderate, or high), healthy diet score (0–1, 2–3, or 4–5) [42], BMI (< 25, 25–30, or ≥ 30 kg/m^2^, defined by WHO [43]).

Further, we performed several sensitivity analyses: (1) only in participants of white ethnicity; (2) excluding case/death events occurring within the first two years of follow-up to reduce potential reverse causation; (3) re-ascertaining the outcomes only using ICD-10 codes; (4) excluding individuals with cancer (ICD-10 code: C00-C97, *N* = 14,866) [44] or all-cause dementia (ICD-10 code: F00-F03, G30, G310, G311, G318, *N* = 38) [45] at recruitment to avoid that these co-existing conditions could confound the results.

#### Exposure and population attributable risk percent

Attributable risk percent (AR%) and population attributable risk percent (PAR%) were calculated to estimate the percentage of decreased cases/deaths if reducing TDI in the population with high TDI (i.e., Q1 vs. Q4). The calculation formulas were

$$\mathrm{AR\% }= \frac{\mathrm{HR }-1}{\mathrm{HR}} \times 100\mathrm{\%},\mathrm{ PAR\% }= \frac{{P}_{e}\left(\mathrm{HR }-1\right)}{{P}_{e}\left(\mathrm{HR }-1\right) +1} \times 100\mathrm{\%}$$

where *P*_e_ denoted as the exposure prevalence with the highest quartile of TDI. The 95% CIs of the AR% and PAR% was calculated using a plug-in method [46].

### Patient and public involvement

The analyses were based on existing data in UK Biobank. To our knowledge, no patients were involved in the design, recruitment, or conduct of the studies, nor did they participate in research question and outcome measures. The study results could not be disseminated to each participant due to deidentification; however, they could obtain the results through broadcasts or science articles.

## Results

### Population characteristics

**Fig. 2:**
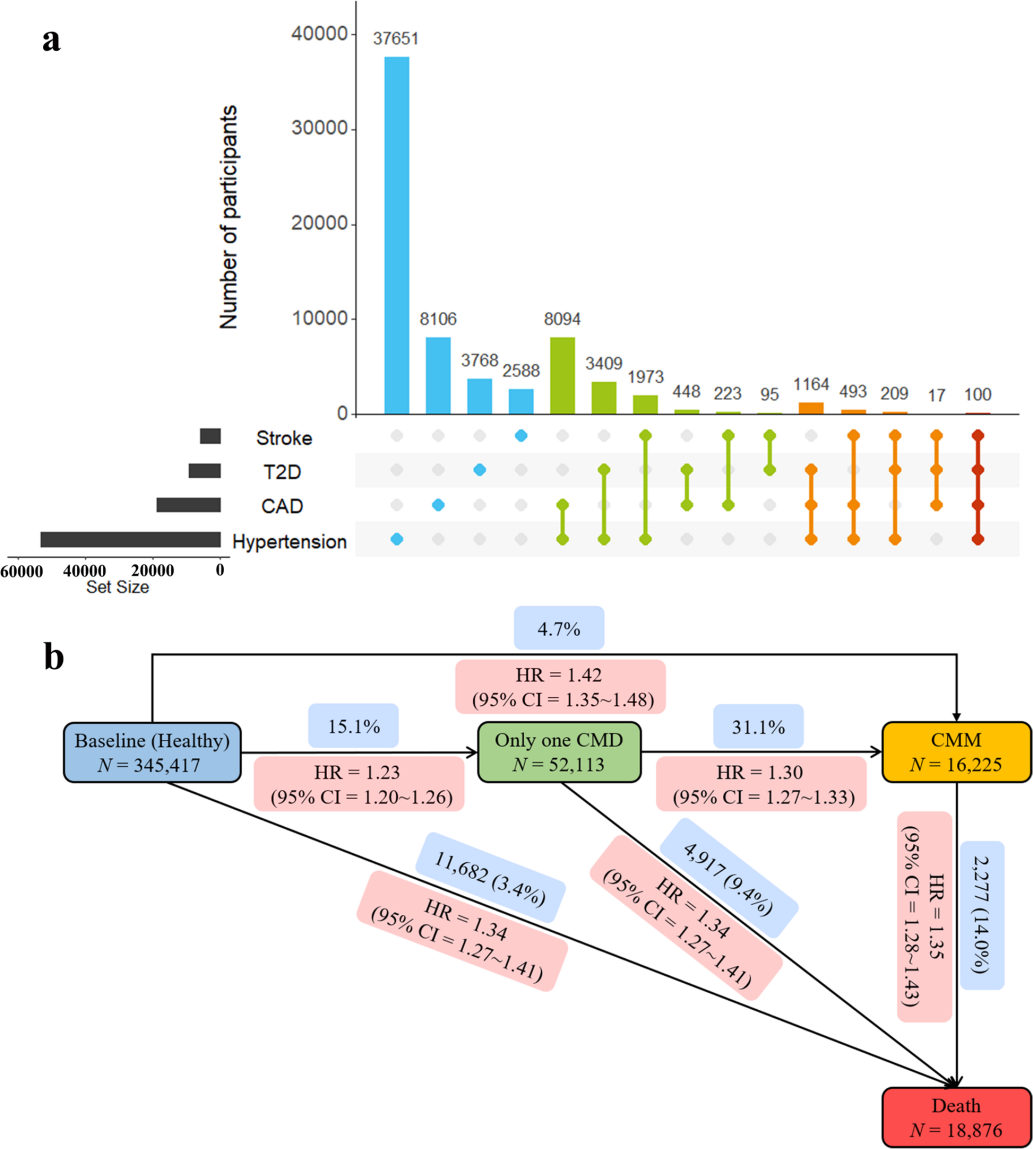
**a** Number of participants for each CMD and different types of CMM (the number of all CMD cases is 217,373); **b** Association of TDI with risks of one CMD, CMM subsequently and death ultimately from different baseline conditions; the HR of TDI for Q4 vs. Q1 and the number of participants under each condition were presented

During a median follow-up of 13.2 years, a total of 68,338 participants developed at least one CMD among those without any CMD at recruitment. Among those participants, 9,210 participants developed T2D (median follow up of 13.4 years), 18,645 developed CAD (median follow up of 13.4 years), 5,698 developed strokes (median follow up of 13.2 years), and 53,093 developed hypertensions (median follow up of 13.3 years). The number of participants for each CMD alone and different statuses of CMM was shown in Fig. 2a. Among those with one CMD, 16,225 further developed CMM (median follow up of 4.8 years). A total of 18,876 deaths were reported during the median follow-up of 13.4 years.

The baseline characteristics of all included participants under different conditions are summarized in Table 1. The correlation coefficients between TDI and other covariates were shown in Supplemental File: Figure S3. Totally, compared to those without any CMD at recruitment, participants with CMD or CMM were more likely to be the older, smoker, and drinker, and had higher BMI, DPB and SBP, lower education and income, less physical activity, and less healthy diet.

**Table 1:** Baseline characteristics of the included participants

covariate	CMD free (*N* = 277,079)	T2D^*^ (*N* = 9210)	CAD^*^ (*N* = 18,645)	stroke^*^ (*N* = 5698)	hypertension^*^ (*N* = 53,093)	CMM^*^ (*N* = 16,225)
TDI	-1.5 (3.0)	-0.4 (3.4)	-1.2 (3.2)	-1.2 (3.2)	-1.1 (3.2)	-0.9 (3.3)
Age at recruitment (years)	54.9 (8.1)	58.1 (7.8)	59.7 (7.3)	60.7 (7.2)	59.4 (7.5)	60.2 (7.2)
BMI (kg/m^2^)	26.3 (4.2)	30.6 (5.5)	27.6 (4.4)	27 (4.5)	28.2 (4.9)	28.8 (5.1)
DBP (mmHg)	79.9 (9.2)	85.4 (10.3)	83.6 (10.2)	84 (10.8)	88.1 (10.6)	86.6 (10.6)
SBP (mmHg)	132.5 (16.4)	143.3 (18.8)	142.3 (18.8)	143.5 (19.8)	150.3 (19.5)	148.2 (19.4)
Male (%)	111,431 (40.2)	4827 (52.4)	11,131 (59.7)	3038 (53.3)	24,878 (46.9)	9343 (57.6)
Ethnicity (%)						
Non-white	13,486 (4.9)	1107 (12.0)	849 (4.6)	209 (3.7)	2898 (5.5)	1065 (6.6)
White	262,191 (94.6)	7971 (86.5)	17,645 (94.6)	5454 (95.7)	49,749 (93.7)	14,982 (92.3)
Missing	1402 (0.5)	132 (1.5)	151 (0.8)	35 (0.6)	446 (0.8)	178 (1.1)
Qualifications (%)						
with college	136,520 (49.3)	4539 (49.3)	8923 (47.9)	2684 (47.1)	25,948 (48.9)	7715 (47.6)
without college	104,863 (37.8)	1975 (21.4)	5059 (27.1)	1569 (27.5)	13,829 (26)	3798 (23.4)
Missing	35,696 (12.9)	2696 (29.3)	4663 (25)	1445 (25.4)	13,316 (25.1)	4712 (29)
Smoking (%)						
No	116,563 (42.1)	3347 (36.3)	6447 (34.6)	2005 (35.2)	20,102 (37.9)	5577 (34.4)
Yes	160,088 (57.8)	5781 (62.8)	12,126 (65)	3679 (64.6)	32,777 (61.7)	10,547 (65)
Missing	428 (0.1)	82 (0.9)	72 (0.4)	14 (0.2)	214 (0.4)	101 (0.6)
Drinking (%)						
No	267,839 (96.7)	8582 (93.2)	17,699 (94.9)	5424 (95.2)	50,618 (95.3)	15,285 (94.2)
Yes	8809 (3.2)	546 (5.9)	874 (4.7)	260 (4.6)	2260 (4.3)	839 (5.2)
Missing	431 (0.1)	82 (0.9)	72 (0.4)	14 (0.2)	215 (0.4)	101 (0.6)
Income (%)						
< £18,000	40,521 (14.6)	2463 (26.7)	4308 (23.1)	1431 (25.1)	12,169 (22.9)	4212 (26.0)
£18,000 ~ £30,999	56,224 (20.3)	2092 (22.7)	4209 (22.6)	1320 (23.2)	12,024 (22.6)	3676 (22.7)
£31,000 ~ £51,999	67,997 (24.5)	1670 (18.1)	3822 (20.5)	1091 (19.1)	10,702 (20.2)	3084 (19.0)
£52,000 ~ £100,000	60,282 (21.8)	958 (10.4)	2560 (13.7)	699 (12.3)	6940 (13.1)	1768 (10.9)
or > £100,000	17,620 (6.4)	216 (2.3)	668 (3.6)	188 (3.3)	1708 (3.2)	407 (2.4)
Missing	34,435 (12.4)	1811 (19.8)	3078 (16.5)	969 (17.0)	9550 (18.0)	3078 (19.0)
Physical activity (%)						
low	39,185 (14.1)	1690 (18.3)	2743 (14.7)	766 (13.4)	7833 (14.8)	2487 (15.3)
moderate	92,387 (33.4)	2617 (28.4)	5635 (30.2)	1715 (30.1)	16,163 (30.4)	4741 (29.2)
high	95,083 (34.3)	2373 (25.8)	6081 (32.6)	1941 (34.1)	15,873 (29.9)	4854 (29.9)
Missing	50,424 (18.2)	2530 (27.6)	4186 (22.5)	1276 (22.4)	13,224 (24.9)	4143 (25.6)
Health diet score (%)						
0	3956 (1.4)	229 (2.5)	378 (2.0)	115 (2.0)	927 (1.7)	364 (2.2)
1	19,431 (7.0)	897 (9.7)	1601 (8.6)	475 (8.3)	4128 (7.8)	1451 (8.9)
2	44,827 (16.2)	1662 (18.0)	3249 (17.4)	1009 (17.7)	8861 (16.7)	2890 (17.8)
3	63,852 (23.0)	2119 (23.0)	4305 (23.1)	1249 (21.9)	12,347 (23.3)	3715 (22.9)
4	65,290 (23.6)	1759 (19.1)	3898 (20.9)	1232 (21.6)	11,617 (21.9)	3224 (19.9)
5	37,671 (13.6)	797 (8.7)	1981 (10.6)	666 (11.7)	6321 (11.9)	1617 (10.0)
Missing	42,052 (15.2)	1747 (19.0)	3233 (17.4)	952 (16.8)	8892 (16.7)	2964 (18.3)

Data were presented as frequency (%) and mean (standard deviation, SD); the asterisk (*) indicates the diagnosis or occurrance during the follow-up

### From baseline to one CMD, CMM and all-cause mortality

For participants without any CMD at baseline, after the adjustment of covariates, TDI was significantly related to elevated risk of developing one CMD (HR = 1.09, 95%CIs = 1.08 ~ 1.10 for per 1 SD increase of TDI) (Table 2), with HR ranging from 1.07 (95%CIs = 1.06 ~ 1.08) for hypertension to 1.19 (95%CIs = 1.16 ~ 1.22) for T2D (Table 3). Compared to participants with the lowest quantile (Q1) of TDI, the HR of those with the highest quantile (Q4) for developing one CMD was 1.23 (95%CIs = 1.20 ~ 1.26), and the HR of Q4 for each single CMD ranged from 1.18 (95%CIs = 1.15 ~ 1.22) for hypertension to 1.52 (95%CIs = 1.41 ~ 1.63) for T2D.

**Table 2:** Association of TDI with one CMD, CMM and all-cause mortality

Association	TDI	HR (95% CIs)	*P*-value
**Healthy → One CMD**	Continuous^*^	1.09 (1.08, 1.10)	**1.10 × 10**^**–96**^
	Q2	1.04 (1.02, 1.07)	**3.28 × 10**^**–4**^
	Q3	1.09 (1.06, 1.11)	**2.01 × 10**^**–13**^
	Q4	1.23 (1.20, 1.26)	**2.49 × 10**^**–70**^
**One CMD → CMM**	Continuous^*^	1.11 (1.10, 1.12)	**3.38 × 10**^**–154**^
	Q2	1.06 (1.04, 1.09)	**2.95 × 10**^**–8**^
	Q3	1.10 (1.08, 1.13)	**1.25 × 10**^**–19**^
	Q4	1.30 (1.27, 1.33)	**5.01 × 10**^**–125**^
**CMM → Death**	Continuous^*^	1.13 (1.11, 1.15)	**5.93 × 10**^**–37**^
	Q2	1.06 (1.01, 1.12)	**2.29 × 10**^**–2**^
	Q3	1.14 (1.09, 1.21)	**4.59 × 10**^**–7**^
	Q4	1.35 (1.28, 1.43)	**2.44 × 10**^**–29**^
**Healthy → CMM**	Continuous^*^	1.16 (1.14, 1.18)	**1.38 × 10**^**–79**^
	Q2	1.04 (1.00, 1.09)	7.20 × 10^–2^
	Q3	1.07 (1.03, 1.13)	**2.74 × 10**^**–3**^
	Q4	1.42 (1.35, 1.48)	**2.63 × 10**^**–50**^
**Healthy → Death**	Continuous^*^	1.15 (1.13, 1.17)	**6.35 × 10**^**–53**^
	Q2	1.01 (0.96, 1.06)	7.96 × 10^–1^
	Q3	1.04 (0.98, 1.09)	1.76 × 10^–1^
	Q4	1.34 (1.27, 1.41)	**2.30 × 10**^**–28**^
**One CMD → Death**	Continuous^*^	1.14 (1.12, 1.16)	**1.68 × 10**^**–43**^
	Q2	1.06 (1.00, 1.11)	**4.62 × 10**^**–2**^
	Q3	1.11 (1.06, 1.17)	**7.15 × 10**^**–5**^
	Q4	1.34 (1.27, 1.41)	**1.48 × 10**^**–27**^

*Q2 *the second quartile, *Q3 *the third quartile, *Q4 *the fourth quartile, *CMD* cardiometabolic disease, *CMM* cardiometabolic multi-morbidity. The asterisk (*) indicates TDI was treated as continuous variable and the HR per SD (3.03) increase of TDI was calculated. The bold *P*-value indicates it is significant at the level of 0.05

**Table 3:** Association of TDI with one CMD in participants without any CMD at baseline, and subsequent CMM and all-cause mortality in CMD cases

Association	without any CMD		with one CMD			with one CMD		
	HR (95%CIs)	*P*-value	Association	HR (95%CIs)	*P*-value	Association	HR (95%CIs)	*P*-value
**Healthy → T2D**			**T2D → CMM**			**T2D → Death**		
Continuous^*^	1.19 (1.16, 1.22)	**9.03 × 10**^**–42**^	Continuous^*^	1.05 (1.03, 1.06)	**1.12 × 10**^**–7**^	Continuous^*^	1.12 (1.08, 1.16)	**1.85 × 10**^**–9**^
Q2	1.07 (1.00, 1.16)	6.36 × 10^–2^	Q2	1.00 (0.97, 1.05)	8.13 × 10^–1^	Q2	1.10 (1.01, 1.20)	**3.56 × 10**^**–2**^
Q3	1.18 (1.10, 1.27)	**1.16 × 10**^**–5**^	Q3	1.05 (1.01, 1.10)	**1.85 × 10**^**–2**^	Q3	1.14 (1.03, 1.25)	**7.45 × 10**^**–3**^
Q4	1.52 (1.41, 1.63)	**4.99 × 10**^**–29**^	Q4	1.11 (1.06, 1.16)	**5.26 × 10**^**–6**^	Q4	1.30 (1.18, 1.43)	**1.82 × 10**^**–7**^
**Healthy → CAD**			**CAD → CMM**			**CAD → Death**		
Continuous^*^	1.08 (1.06, 1.10)	**4.69 × 10**^**–15**^	Continuous^*^	1.03 (1.01, 1.04)	**1.29 × 10**^**–4**^	Continuous^*^	1.10 (1.07, 1.13)	**2.19 × 10**^**–11**^
Q2	1.01 (0.96, 1.06)	7.40 × 10^–1^	Q2	1.01 (0.97, 1.04)	7.29 × 10^–1^	Q2	1.11 (1.03, 1.20)	**3.81 × 10**^**–3**^
Q3	1.08 (1.03, 1.13)	**2.46 × 10**^**–3**^	Q3	1.00 (0.97, 1.04)	8.03 × 10^–1^	Q3	1.08 (1.00, 1.17)	**4.09 × 10**^**–2**^
Q4	1.19 (1.13, 1.25)	**2.30 × 10**^**–11**^	Q4	1.07 (1.03, 1.11)	**1.34 × 10**^**–4**^	Q4	1.30 (1.02, 1.40)	**7.67 × 10**^**–11**^
**Healthy → stroke**			**Stroke → CMM**			**Stroke → Death**		
Continuous^*^	1.11 (1.08, 1.15)	**1.28 × 10**^**–10**^	Continuous^*^	1.03 (1.01, 1.06)	**1.02 × 10**^**–2**^	Continuous^*^	1.08 (1.04, 1.12)	**1.19 × 10**^**–4**^
Q2	1.04 (0.95, 1.13)	3.93 × 10^–1^	Q2	1.00 (0.94, 1.06)	9.63 × 10^–1^	Q2	0.97 (0.88, 1.07)	6.09 × 10^–1^
Q3	1.08 (0.99, 1.17)	8.51 × 10^–2^	Q3	1.04 (0.98, 1.11)	1.61 × 10^–1^	Q3	1.01 (0.91, 1.12)	8.38 × 10^–1^
Q4	1.29 (1.18, 1.41)	**1.49 × 10**^**–8**^	Q4	1.07 (1.00, 1.15)	**4.14 × 10**^**–2**^	Q4	1.17 (1.06, 1.31)	**3.21 × 10**^**–3**^
**Healthy → hypertension**			**Hypertension → CMM**			**Hypertension → Death**		
Continuous^*^	1.07 (1.06, 1.08)	**1.12 × 10**^**–33**^	Continuous^*^	1.09 (1.08, 1.10)	**9.79 × 10**^**–63**^	Continuous^*^	1.13 (1.11, 1.16)	**8.58 × 10**^**–42**^
Q2	1.04 (1.01, 1.07)	**8.03 × 10**^**–3**^	Q2	1.05 (1.02, 1.08)	**2.77 × 10**^**–4**^	Q2	1.09 (1.04, 1.14)	**5.14 × 10**^**–4**^
Q3	1.07 (1.04, 1.11)	**1.41 × 10**^**–6**^	Q3	1.07 (1.04, 1.10)	**1.25 × 10**^**–7**^	Q3	1.16 (1.11, 1.22)	**1.24 × 10**^**–9**^
Q4	1.18 (1.15, 1.22)	**5.46 × 10**^**–28**^	Q4	1.24 (1.21, 1.28)	**1.78 × 10**^**–55**^	Q4	1.36 (1.29, 1.43)	**6.13 × 10**^**–33**^

*Q2 *the second quantile; *Q3 *the third quantile, *Q4 *the fourth quantile, *CMD* cardiometabolic disease, *CMM* cardiometabolic multi-morbidity. The asterisk (*) indicates that TDI was treated as continuous variable and the HR per SD increase of TDI was calculated. The bold *P*-value indicates it is significant at the level of 0.05

For participants without any CMD at baseline, after adjusting for covariates, we discovered that per 1 SD increase of TDI could lead to 16% (95%CIs = 14% ~ 18%) higher risk of developing CMM or 15% (95%CIs = 13% ~ 17%) larger risk of all-cause mortality, respectively, with the HR of Q4 being 1.42 (95%CIs = 1.35 ~ 1.48) and 1.34 (95%CIs = 1.27 ~ 1.4122), respectively. Meanwhile, an incremental trend of HR from Q1 to Q4 was observed for each association scenario (Table 2).

### From one CMD to CMM and all-cause mortality

For participants with one CMD during the follow up, after controlling for covariates, we observed that per 1 SD increase of TDI would result in 11% (95%CIs = 10% ~ 12%) higher risk of CMM (median follow up of 4.8 years) and 14% (95%CIs = 12% ~ 16%) higher risk of all-cause mortality (median follow up of 5.4 years) (Table 2), with the HR of Q4 equal to 1.30 (95%CIs = 1.27 ~ 1.33) for CMM and 1.34 (95%CIs = 1.27 ~ 1.41) for all-cause mortality compared to Q1, respectively. For each CMD, the HR of TDI for developing CMM was 1.05 (95%CIs = 1.03 ~ 1.06) for T2D, 1.03 (95%CIs = 1.01 ~ 1.04) for CAD, 1.03 (95%CIs = 1.01 ~ 1.06) for stroke, and 1.09 (95%CIs = 1.08 ~ 1.10) for hypertension; and the HR of all-cause mortality was 1.12 (95%CIs = 1.08 ~ 1.16) for T2D, 1.10 (95%CIs = 1.07 ~ 1.13) for CAD, 1.08 (95%CIs = 1.04 ~ 1.12) for stroke, and 1.13 (95%CIs = 1.11 ~ 1.16) for hypertension (Table 3).

Again, for each CMD, compared to participants with Q1 of TDI, the HR of participants with Q4 for developing CMM or all-cause death was much larger than that of TDI when it was treated as a continuous variable, and an increased trend of HR from Q1 to Q4 was seen for every disease (Table 3). In addition, we found that, compared to Q1, the HRs of Q4 were similar among participants with T2D (HR = 1.11, 95%CIs = 1.06 ~ 1.16), CAD (HR = 1.07, 95%CIs = 1.03 ~ 1.11) and stroke (HR = 1.07, 95%CIs = 1.00 ~ 1.15) for developing CMM, but seemed more pronounced among participants with hypertension (HR = 1.24, 95%CIs = 1.21 ~ 1.28) (Table 3).

### From CMM to all-cause mortality

During the median follow up of 4.5 years, after adjusting for covariates, we identified that per 1 SD increase of TDI could result in 13% (95%CIs = 11% ~ 15%) higher risk of death among participants with CMM, with the HR of Q4 equal to 1.35 (95%CIs = 1.28 ~ 1.43) compared to Q1 (Table 2). Finally, for ease of comparison, we demonstrated some estimated associations in Fig. 2b.

### Subgroup and sensitive analysis

Subgroup analyses were conducted among different covariate levels (Supplemental File: Figures S4-S12). We discovered that increased TDI was associated with higher risk of developing one CMD, or causing CMM and death in nearly all subgroups. Moreover, it was shown that participants smoking and drinking previously, without college/university degree, with lower income, lower physical activity, and lower healthy diet score in general had higher risk.

In addition, we identified that those discovered associations remained significant if only analyzing participants of white ethnicity (Supplemental File: Figure S13), and observed consistent association patterns between TDI and each disease in all association scenarios after excluding events occurred within the first two years of follow-up (Supplemental File: Figure S14). More interestingly, nearly all estimated effects became stronger in this analysis (an average of 6.6% increase in HR), implying that TDI likely had a more evident long-term role in developing those diseases and leading to death. Moreover, while only using ICD-10 coding to more specialize the outcomes (Supplemental File: Figure S15) and excluding individuals suffering from cancer or all-cause dementia at the time of recruitment (Supplemental File: Figure S16), the role of TDI has not changed substantially in almost all transition stages of CMM trajectory.

### Estimated AR% and PAR%

Compared to Q1, the AR% for Q4 of TDI ranged from 6.5% (from CAD baseline to CMM or from stroke baseline to CMM) to 37.5% (from health baseline to T2D), and the PAR% ranged from 1.7% (from CAD baseline to CMM or from stroke baseline to CMM) to 13.0% (from health baseline to T2D) (Supplemental File: Table S2). For example, 37.5% of incident T2D cases with high deprived condition could be attributable to TDI, and 13.0% of incident T2D cases in the total population could be attributable to TDI.

## Discussions

### Summary of our work

In this study, we have comprehensively examined the role of TDI in all disease transition stages from baseline to single CMD, subsequently to CMM, and ultimately to death. We confirmed the positive association between TDI and each CMD, and further revealed that elevated TDI was associated with greater risk of CMM and all-cause mortality among participants with one CMD at baseline. We also discovered that increased TDI was also related to higher risk of all-cause mortality among participants suffering from CMM.

### Comparison with previous studies

Our current findings of association between TDI and individual CMDs were in agreement with previous research [13, 20, 23, 29]. Cohort studies reported that social deprivation was significantly associated with T2D [13, 17, 18], ischemic stroke [23, 25, 26], and hypertension [29]. In addition, compared to the lowest neighborhood deprivation, Chamberlain et al. observed that the highest neighborhood deprivation was associated with 1.71 (95%CIs = 1.52 ~ 1.94), 1.88 (95%CIs = 1.59 ~ 2.22), 1.73 (95%CIs = 1.40 ~ 2.13), 2.57 (95%CIs = 2.15 ~ 3.06) higher risk of developing diabetes, CAD, stroke and hypertension, respectively [20], which showed stronger associations compared to our findings. Our analysis also provided evidence for the positive association between TDI and all-cause mortality, which was a little stronger than a previous finding (HR = 1.42 *vs*. HR = 1.24) [47].

### Public health implication

Besides revealing that TDI was an independent risk factor of CMM and all-cause mortality, our work is also of sociological importance. It has been well-documented that socioeconomic disadvantage has a pronounced influence on human health outcomes [48–50], and that people with advantaged socioeconomic conditions live longer, and have better mental and physical health compared to those from more deprived environments [51, 52]. Therefore, understanding the connection between TDI and the course from CMD to CMM or mortality has the potential to be instructive for developing efficient prevention strategies, helping minimize disease/mortality risk, reducing medical economic burden, and improving quality of life. As a common indicator quantifying socioeconomic status, TDI is useful for studying health inequalities [53], and can be applied to screen for high-risk populations. Moreover, inspiration could be obtained from the results that immediate intervention of targeting highly deprived individuals would reduce the threat of CMD, CMM, and premature mortality. For example, if individuals' TDI could be reduced from Q4 level to Q1 level, 37.5% of incident T2D cases, 21.9% of incident CAD cases, 28.6% of incident stroke cases or 28.6% of incident hypertension cases would be likely avoided among those free of CMD at baseline.

### Strength, limitations, and future research

The major strength of our study is that large-scale samples and long follow-up periods enabled us to evaluate the role of TDI systematically and thoroughly during the whole progress from baseline to CMM and death. In addition, our work was among the first to survey the role of TDI in the progression trajectory of CMM.

There are several limitations to this study. First, our findings could be vulnerable to selection bias, which has been extensively discussed in previous papers [54–56]. It has been reported that UK Biobank participants were more likely to be white British people [33], lived in less socioeconomically deprived areas, and be more health-conscious [55], which may result in an underestimate of the association. Therefore, caution should be made when extending our findings to general populations.

Second, although we made efforts to control for various common potential confounders associated with cardiovascular disease and diabetes, it is essential to recognize that there might be other residual and unmeasured confounders that were not accounted for but would affect the disease outcomes in our study [57], such as familial predisposition [58], which was not available in our study.

Third, TDI was generated from aggregated data related to the postal region, which is not measured at the individual level and may not completely reflect the differences in actual individual socioeconomic deprivation [11, 59]. On the one hand, besides TDI, there also exist many other indicators of social deprivation like indices of multiple deprivation (IMD) [60], area deprivation index (ADI) [61], social deprivation index (SDI) [62], or newly calculated indicators according to local conditions such as German index of multiple deprivation (GIMD) [17] and EPICES score [24]. On the other hand, individual level of socioeconomic status (SES) based on indicators like income, education and employment was constructed to provide a more detailed and nuanced understanding of individual socioeconomic disparities [31, 63]. Therefore, caution should be used while interpreting these associations, and one of the future directions is to explore influences of other social deprivation measurements on CMM and mortality.

Fourth, non-CMD comorbidities such as cancer and dementia were not adjusted for in our models, however, these co-existing conditions could confound the results [64, 65]. To examine this issue, we have also conducted a sensitivity analysis by excluding individuals with cancer or all-cause dementia at recruitment, but observed no significant changes in associations. In spite of this, more non-CMD comorbidities that could be confounders, such as renal dysfunction [66] and respiratory comorbidities [67], should be considered in future studies.

Finally, our study was conducted as an observational population study, which limited our ability to make a conclusion about the causality between TDI and CMM. Further studies with multiple design strategies are required to confirm our findings and provide a more comprehensive understanding of the potential mechanism underlying the associations observed in the present study.

## Conclusions

We provided epidemiological evidence about the adverse influence of TDI on each CMD, and further revealed that people with one CMD would suffer from higher hazard of CMM and all-cause mortality, and that people with CMM would encounter increased risk of all-cause mortality if living with high deprived conditions.

### Supplementary Information

**Additional file 1: Supplementary information 1.** Treatment for covariates. **Supplementary Table S1.** Definitions of the four cardiometabolic diseases. **Supplementary Table S2.** Attributable risk percent (AR%) and population attributable risk percent (PAR%) of one CMD, CMM and all-cause mortality from different baseline conditions. **Supplementary Figure S1. **Restricted cubic spline (RCS) curves for the association of TDI with one CMD, CMM and all-cause mortality. **Supplementary Figure S2.** Cumulative risk curves for the association of TDI with one CMD, CMM and all-cause mortality. **Supplementary Figure S3. **Correlation coefficients between TDI and other covariates. **Supplementary Figure S4.** Subgroup analyses by sex group for the association of Townsend deprivation index with one CMD, CMM and all-cause mortality from different baseline conditions. **Supplementary Figure S5.** Subgroup analyses by age group for the association of Townsend deprivation index with one CMD, CMM and all-cause mortality from different baseline conditions. **Supplementary Figure S6. **Subgroup analyses by income group for the association of Townsend deprivation index with one CMD, CMM and all-cause mortality from different baseline conditions. **Supplementary Figure S7.** Subgroup analyses by education background for the association of Townsend deprivation index with one CMD, CMM and all-cause mortality from different baseline conditions. **Supplementary Figure S8.** Subgroup analyses by smoking status for the association of Townsend deprivation index with one CMD, CMM and all-cause mortality from different baseline conditions. **Supplementary Figure S9. **Subgroup analyses by drinking status for the association of Townsend deprivation index with one CMD, CMM and all-cause mortality from different baseline conditions. **Supplementary Figure S10. **Subgroup analyses by physical activity for the association of Townsend deprivation index with one CMD, CMM and all-cause mortality from different baseline conditions. **Supplementary Figure S11. **Subgroup analyses by healthy diet score for the association of Townsend deprivation index with one CMD, CMM and all-cause mortality from different baseline conditions. **Supplementary Figure S12.** Subgroup analyses by BMI for the association of Townsend deprivation index with one CMD, CMM and all-cause mortality from different baseline conditions.** Supplementary Figure S13. **Analyses in white ethnicity for the association of Townsend deprivation index with one CMD, CMM and all-cause mortality from different baseline conditions. **Supplementary Figure S14.** Sensitivity analyses for the association of Townsend deprivation index with one CMD, CMM and all-cause mortality from different baseline conditions while excluding events occurred within the first two years of follow-up. **Supplementary Figure S15.** Sensitivity analyses for the association of Townsend deprivation index with one CMD, CMM and all-cause mortality from different baseline conditions only according to ICD-10 codes. **Supplementary Figure S16.** Sensitivity analyses for the association of Townsend deprivation index with one CMD, CMM and all-cause mortality from different baseline conditions while excluding individuals with cancer or dementia at recruitment.

## Data Availability

This study used the UK Biobank resource with the application ID 88159. Researchers can access to the UK Biobank dataset by applying to the UK Biobank official website (https://www.ukbiobank.ac.uk/). All data generated or analyzed during this study are included in this published article and its supplementary information files.
