# Supplementary material for: Influence of social deprivation on morbidity and all-cause mortality of cardiometabolic multi-morbidity: a cohort analysis of the UK Biobank cohort
Source: BMC Public Health. 2023 Nov 7;23:2177. doi: 10.1186/s12889-023-17008-5 (PMC10629082; doi:10.1186/s12889-023-17008-5)
Supplement: Supplementary file 1 — Additional file 1: Supplementary information 1. Treatment for covariates. Supplementary Table S1. Definitions of the four cardiometabolic diseases. Supplementary Table S2. Attributable risk percent (AR%) and population attributable risk percent (PAR%) of one CMD, CMM and all-cause mortality from different baseline conditions. Supplementary Figure S1. Restricted cubic spline (RCS) curves for the association of TDI with one CMD, CMM and all-cause mortality. Supplementary Figure S2. Cumulative risk curves for the association of TDI with one CMD, CMM and all-cause mortality. Supplementary Figure S3. Correlation coefficients between TDI and other covariates. Supplementary Figure S4. Subgroup analyses by sex group for the association of Townsend deprivation index with one CMD, CMM and all-cause mortality from different baseline conditions. Supplementary Figure S5. Subgroup analyses by age group for the association of Townsend deprivation index with one CMD, CMM and all-cause mortality from different baseline conditions. Supplementary Figure S6. Subgroup analyses by income group for the association of Townsend deprivation index with one CMD, CMM and all-cause mortality from different baseline conditions. Supplementary Figure S7. Subgroup analyses by education background for the association of Townsend deprivation index with one CMD, CMM and all-cause mortality from different baseline conditions. Supplementary Figure S8. Subgroup analyses by smoking status for the association of Townsend deprivation index with one CMD, CMM and all-cause mortality from different baseline conditions. Supplementary Figure S9. Subgroup analyses by drinking status for the association of Townsend deprivation index with one CMD, CMM and all-cause mortality from different baseline conditions. Supplementary Figure S10. Subgroup analyses by physical activity for the association of Townsend deprivation index with one CMD, CMM and all-cause mortality from different baseline conditions. Supplementary Fi [file 12889_2023_17008_MOESM1_ESM.docx]

**Supplementary File**

**Treatment for covariates**

We categorized these covariates into sociodemographic characteristics and lifestyle factors. Particularly, sociodemographic characteristics included age (continuous), sex (men and women), ethnicity (white and non-white), education (with or without college/university degree), and income (<£18,000, £18,000~£30,999, £31,000~£51,999, £52,000~£100,000, or >£100,000). Lifestyle factors were measured by self-report, including smoking status (former smoking, and former non-smoking) and alcohol drinking status (former drinking, and former non-drinking). Physical activity was evaluated via the International Physical Activity Questionnaire (IPAQ) short form [[1](#_ENREF_1)], based on which physical activity was divided into three groups: low (<600 min/week), moderate (between 600 and 3000 min/week), and high (>3000 min/week). In addition, healthy diet score was calculated in terms of daily diet factors and ranged from 0 to 5 [[2](#_ENREF_2)]. One point was assigned when met each of the following favorable conditions: (1) vegetable intake ≥four table-spoons/day; (2) fruit intake ≥three pieces/day; (3) fish intake ≥twice/week; (4) unprocessed red meat intake ≤twice/week; (5) and processed meat intake ≤twice/week. Finally, we took the summation of each point as the total healthy diet score.

Furthermore, height, weight, diastolic blood pressure (DBP), and systolic blood pressure (SBP) were measured by trained nurses using standardized procedures during the initial assessment center visit. SBP or DBP was calculated as the average of blood pressure readings when both automated and manual measurements could be obtained. In addition, considering that medication for blood pressure may lead to the decrease in both SBP and DBP and thus biased the relationship, we added 15mmHg for SBP and 10mHg for DBP respectively for participants who received the special medication for blood pressure (Fields 6153 and 6177) [[3](#_ENREF_3)].

Table S1. Definitions of the four cardiometabolic diseases

|  | **Fields** | **Codes** |
| --- | --- | --- |
| Type II diabetes | 41270 | E11 |
|  | 20002 | 1223 |
|  | 2443 | 1 |
|  | 6153 | 3 |
|  | 6157 | 3 |
|  | 2976 | - |
| Coronary Artery Disease | 41270 | I20, I21, I22, I23, I24, I25 |
|  | 20002 | 1066, 1074, 1075 |
|  | 6150 | 1,2 |
|  | 3627 | - |
|  | 3894 | - |
| Stroke | 41270 | I60, I61, I62, I63, I64, I69 |
|  | 20002 | 1081, 1086, 1491, 1583 |
|  | 6150 | 3 |
|  | 4056 | - |
|  | 42006 | - |
|  | 42008 | - |
|  | 42010 | - |
|  | 42012 | - |
| Hypertension | 41270 | I10, I11, I12, I13, I14, I15 |
|  | 20002 | 1065, 1072, 1073 |
|  | 6150 | 4 |
|  | 6153 | 2 |
|  | 6177 | 2 |
|  | 2966 | - |

Table S2. Attributable risk percent (AR%) and population attributable risk percent (PAR%) of one CMD, CMM and all-cause mortality from different baseline conditions.

| Association | AR% (95%CIs) | PAR% (95%CIs) |
| --- | --- | --- |
| **Healthy → One CMD** | 18.7 (16.7, 20.6) | 5.4 (4.8, 6.1) |
| Health baseline → T2D | 37.5 (33.8, 41.5) | 13.0 (11.3, 15.1) |
| Health baseline → CAD | 21.9 (18.0, 24.8) | 6.5 (5.2, 7.6) |
| Health baseline→ stroke | 28.6 (23.1, 33.8) | 9.1 (7.0, 11.3) |
| Healthy baseline → hypertension | 19.4 (17.4, 21.9) | 5.7 (5.0, 6.5) |
| **Healthy → CMM** | 29.6 (25.9, 32.4) | 9.5 (8.1, 10.7) |
| **Healthy → Death** | 25.4 (21.3, 29.1) | 7.8 (6.3, 9.3) |
| **one CMD → CMM** | 23.1 (21.3, 24.8) | 7.0 (6.3, 7.6) |
| T2D baseline → CMM | 7.4 (3.8, 10.7) | 2.0 (1.0, 2.9) |
| CAD baseline → CMM | 6.5 (3.8, 9.1) | 1.7 (1.0, 2.4) |
| stroke baseline → CMM | 6.5 (2.0, 11.5) | 1.7 (0.5, 3.1) |
| hypertension baseline → CMM | 23.7 (21.9, 25.4) | 7.2 (6.5, 7.8) |
| **one CMD → Death** | 25.4 (21.3, 29.1) | 7.8 (6.3, 9.3) |
| T2D baseline → Death | 29.1 (23.7, 34.2) | 9.3 (7.2, 11.5) |
| CAD baseline → Death | 27.5 (23.1, 32.0) | 8.7 (7.0, 10.5) |
| stroke baseline → Death | 18.0 (10.7, 24.8) | 5.2 (2.9, 7.6) |
| hypertension baseline → Death | 32.0 (29.6, 34.6) | 10.5 (9.5, 11.7) |
| **CMM → Death** | 25.9 (21.9, 30.1) | 8.1 (6.5, 9.7) |

Note: CMD, cardiometabolic disease; CMM, cardiometabolic multi-morbidity. Both of AR% and PAR% correspond to the hazard ratios of the fourth quartile (Q4) compared to the first quartile (Q1).


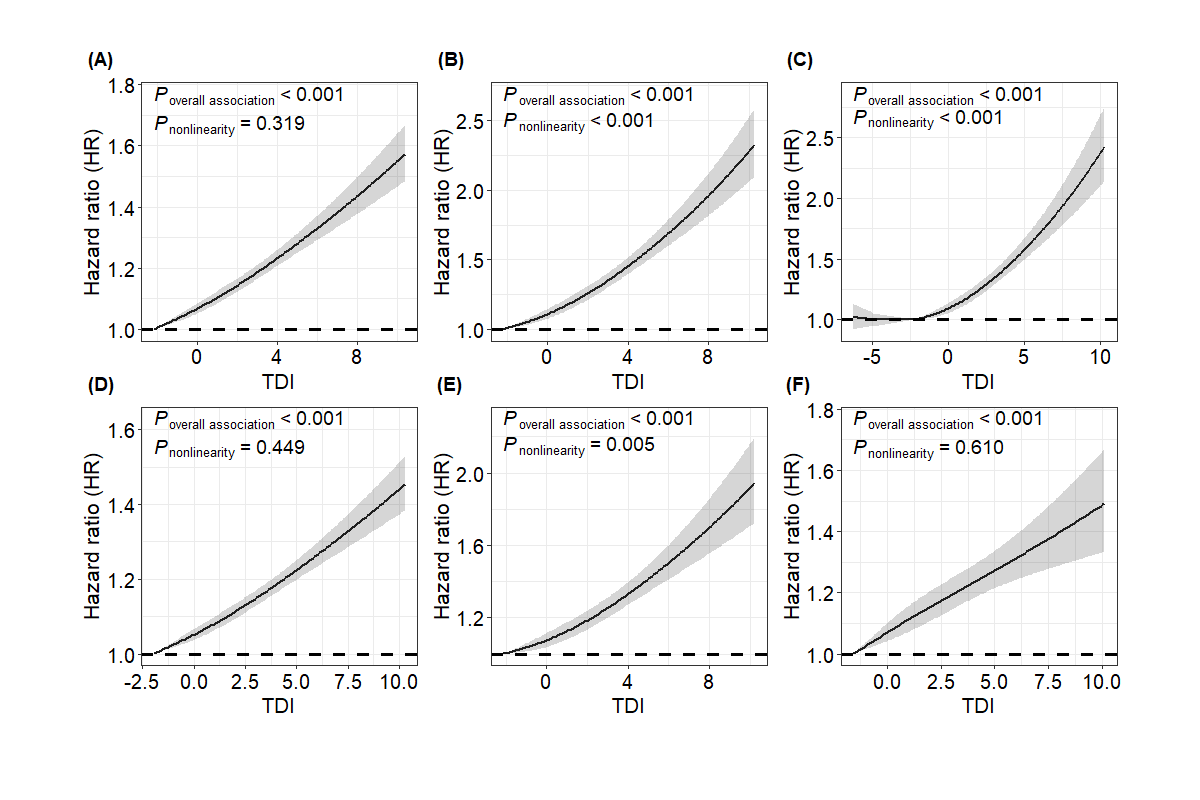


Figure S1. Restricted cubic spline (RCS) curves for the association of TDI with one CMD, CMM and all-cause mortality. (A) healthy to one CMD; (B) healthy to CMM; (C) healthy to all-cause mortality; (D) one CMD to CMM; (E) one CMD to all-cause mortality; (F) CMM to all-cause mortality. Abbreviation: TDI, Townsend deprivation index; CMD: cardiometabolic disease; CMM, cardiometabolic multi-morbidity.


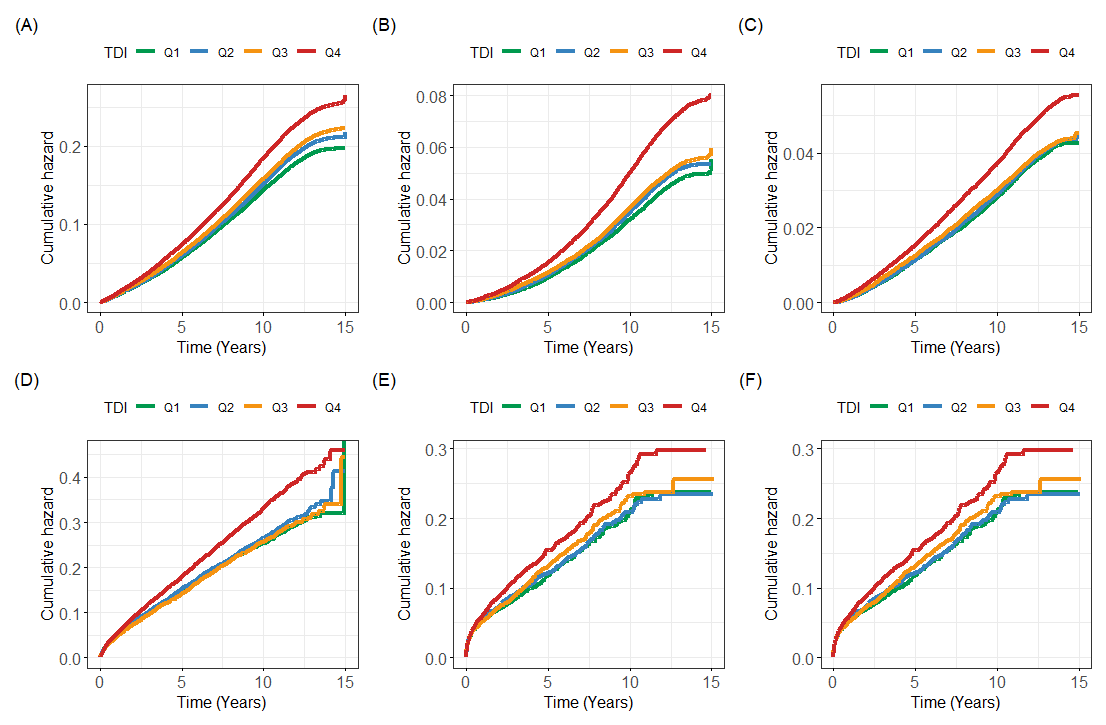


Figure S2. Cumulative risk curves for the association of TDI with one CMD, CMM and all-cause mortality. (A) healthy to one CMD; (B) healthy to CMM; (C) healthy to all-cause mortality; (D) one CMD to CMM; (E) one CMD to all-cause mortality; (F) CMM to all-cause mortality. Abbreviation: TDI, Townsend deprivation index; CMD: cardiometabolic disease; CMM, cardiometabolic multi-morbidity.


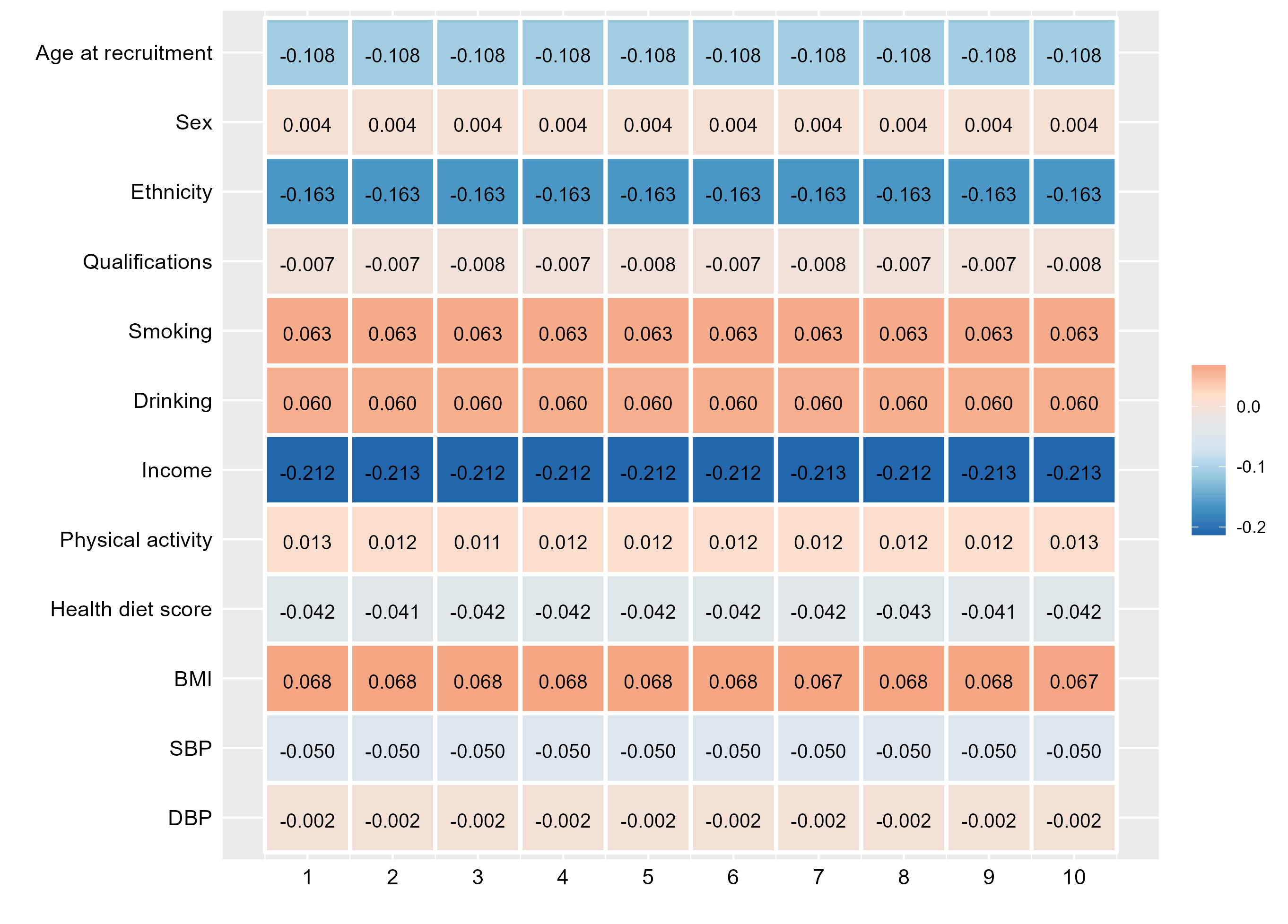


Figure S3. Correlation coefficients between TDI and other covariates. Note: The horizontal axis represents the number of ten imputed datasets and the vertical axis represents other covariates. The numbers in the square box represent correlation coefficient between TDI and other covariates (Pearson coefficient for “age at recruitment”, “BMI”, “SBP” and “DBP”; and Spearman coefficient for remaining covariates).


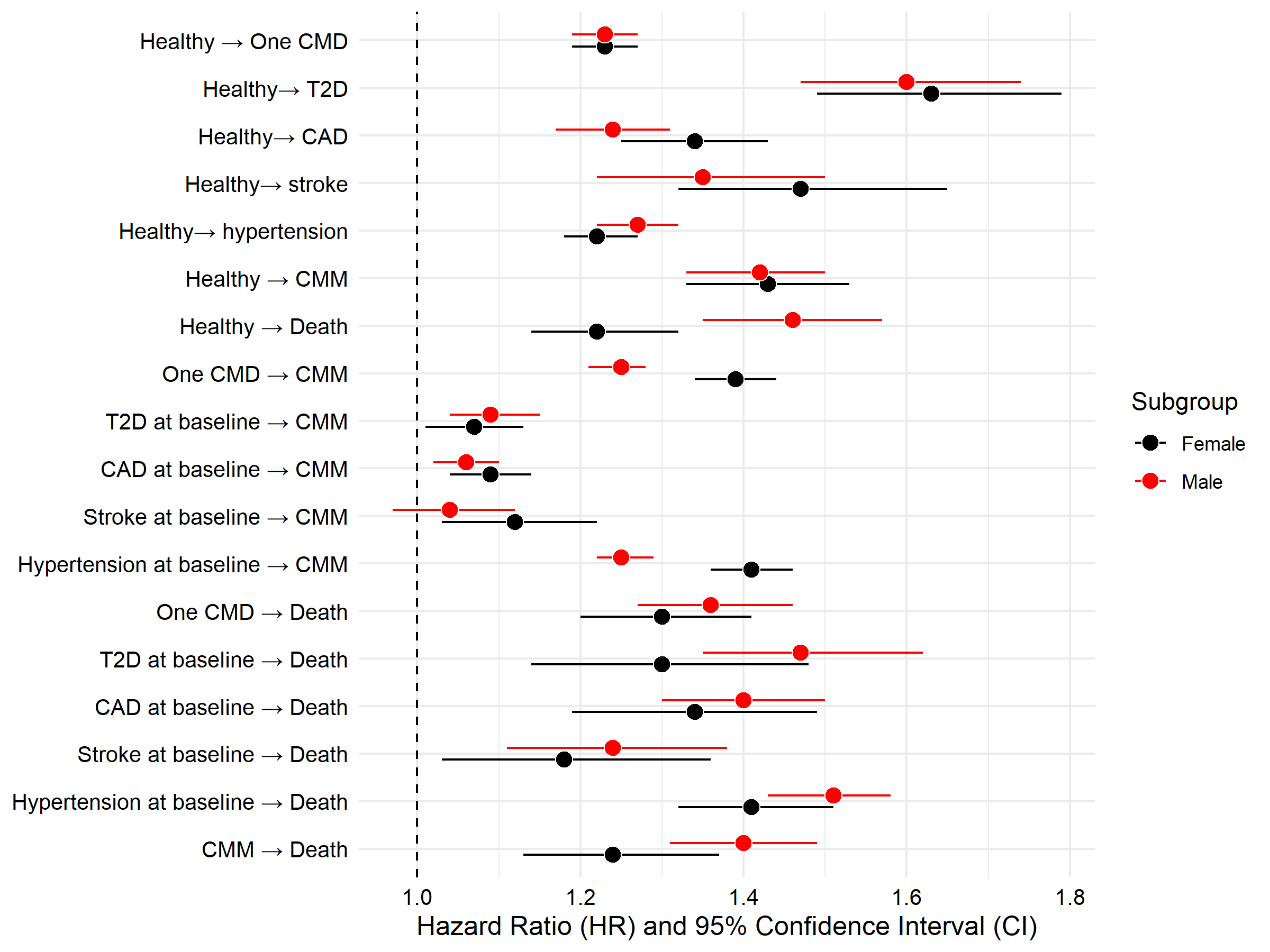


Figure S4. Subgroup analyses by sex group for the association of Townsend deprivation index with one CMD, CMM and all-cause mortality from different baseline conditions. Note: CMD: cardiometabolic disease; CMM, cardiometabolic multi-morbidity. The HR values correspond to the hazard ratios of the fourth quartile (Q4) compared to the first quartile (Q1).


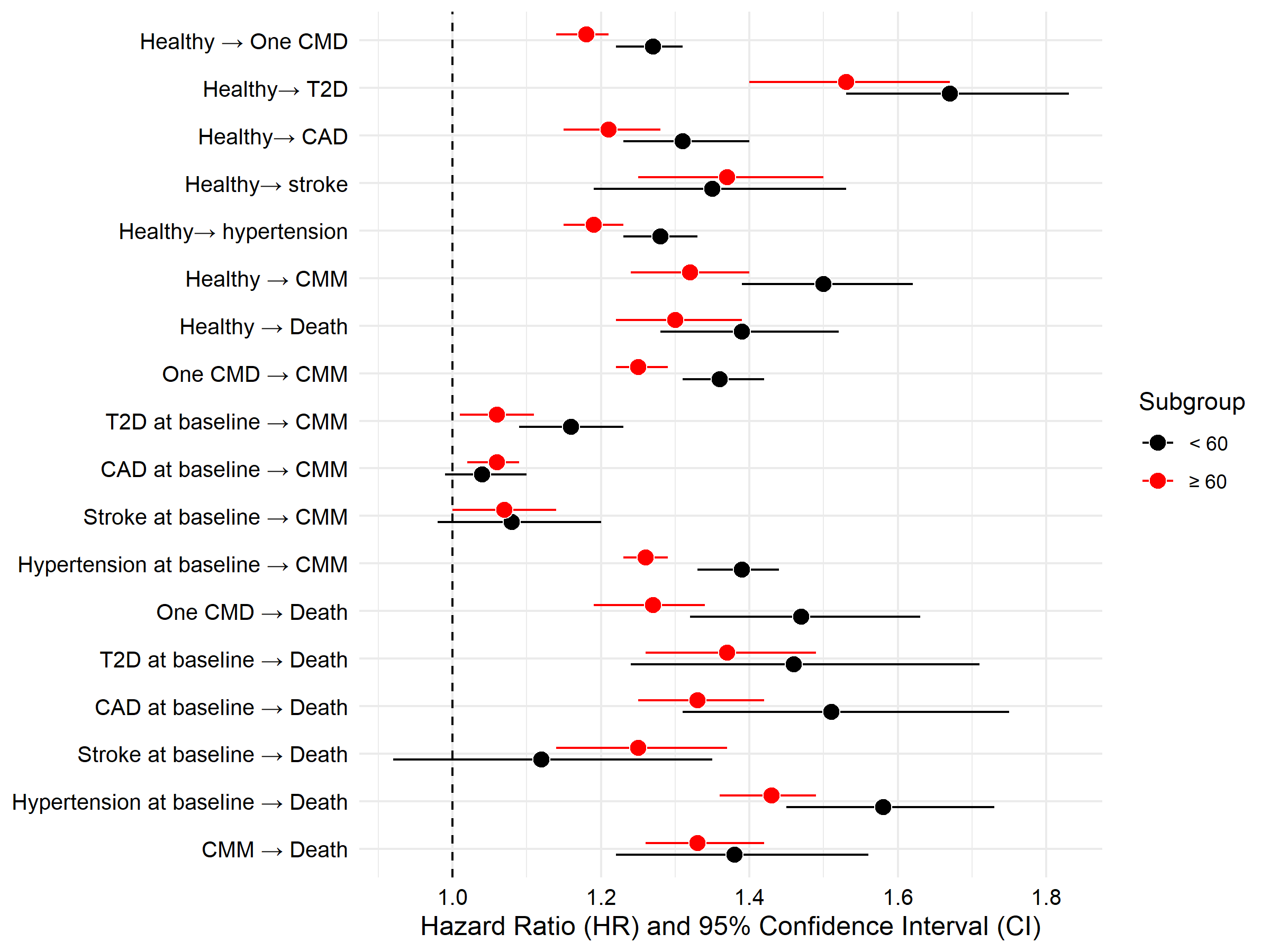


Figure S5. Subgroup analyses by age group for the association of Townsend deprivation index with one CMD, CMM and all-cause mortality from different baseline conditions. Note: CMD: cardiometabolic disease; CMM, cardiometabolic multi-morbidity. The HR values correspond to the hazard ratios of the fourth quartile (Q4) compared to the first quartile (Q1).


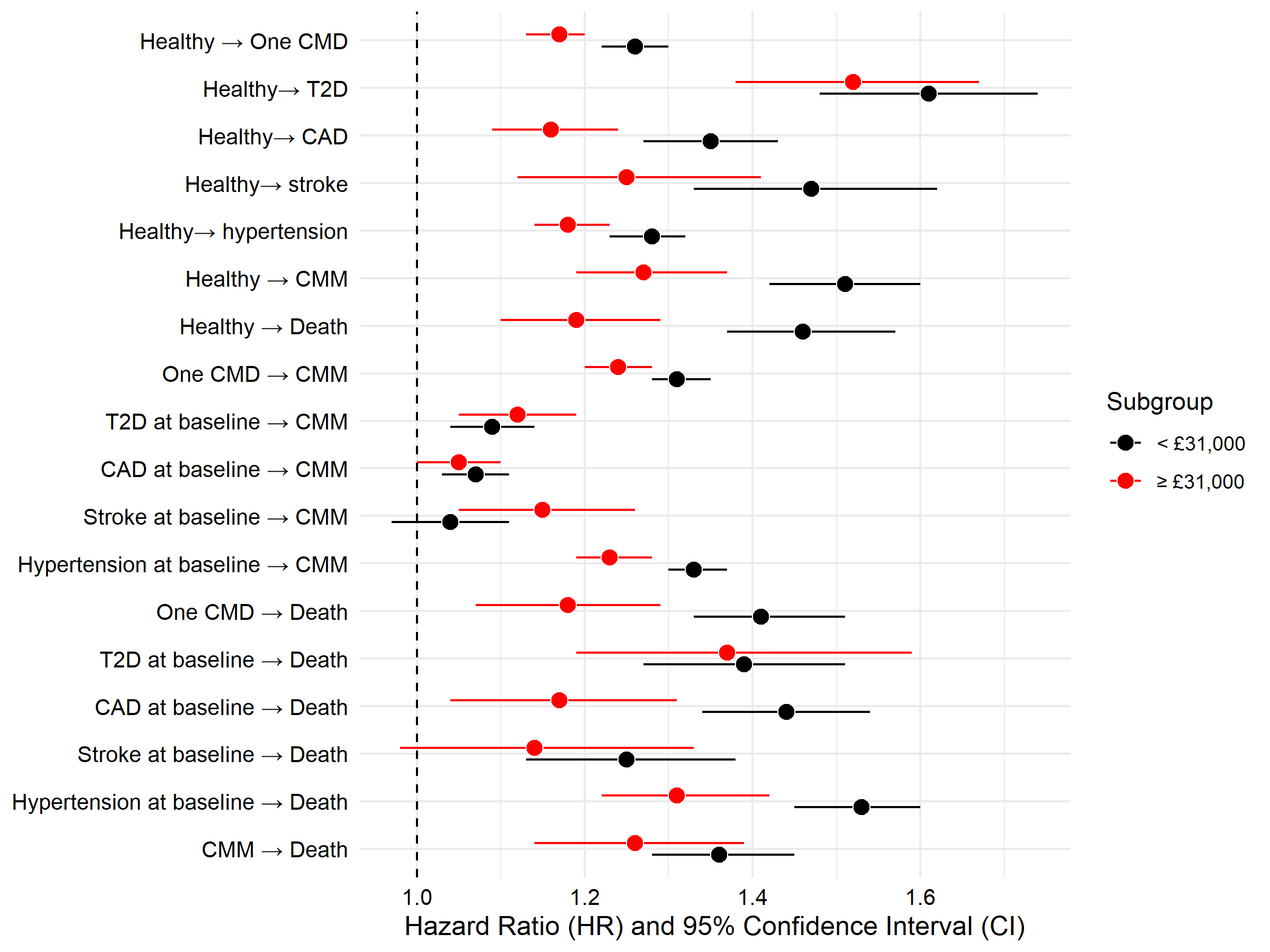


Figure S6. Subgroup analyses by income group for the association of Townsend deprivation index with one CMD, CMM and all-cause mortality from different baseline conditions. Note: CMD: cardiometabolic disease; CMM, cardiometabolic multi-morbidity. The HR values correspond to the hazard ratios of the fourth quartile (Q4) compared to the first quartile (Q1).


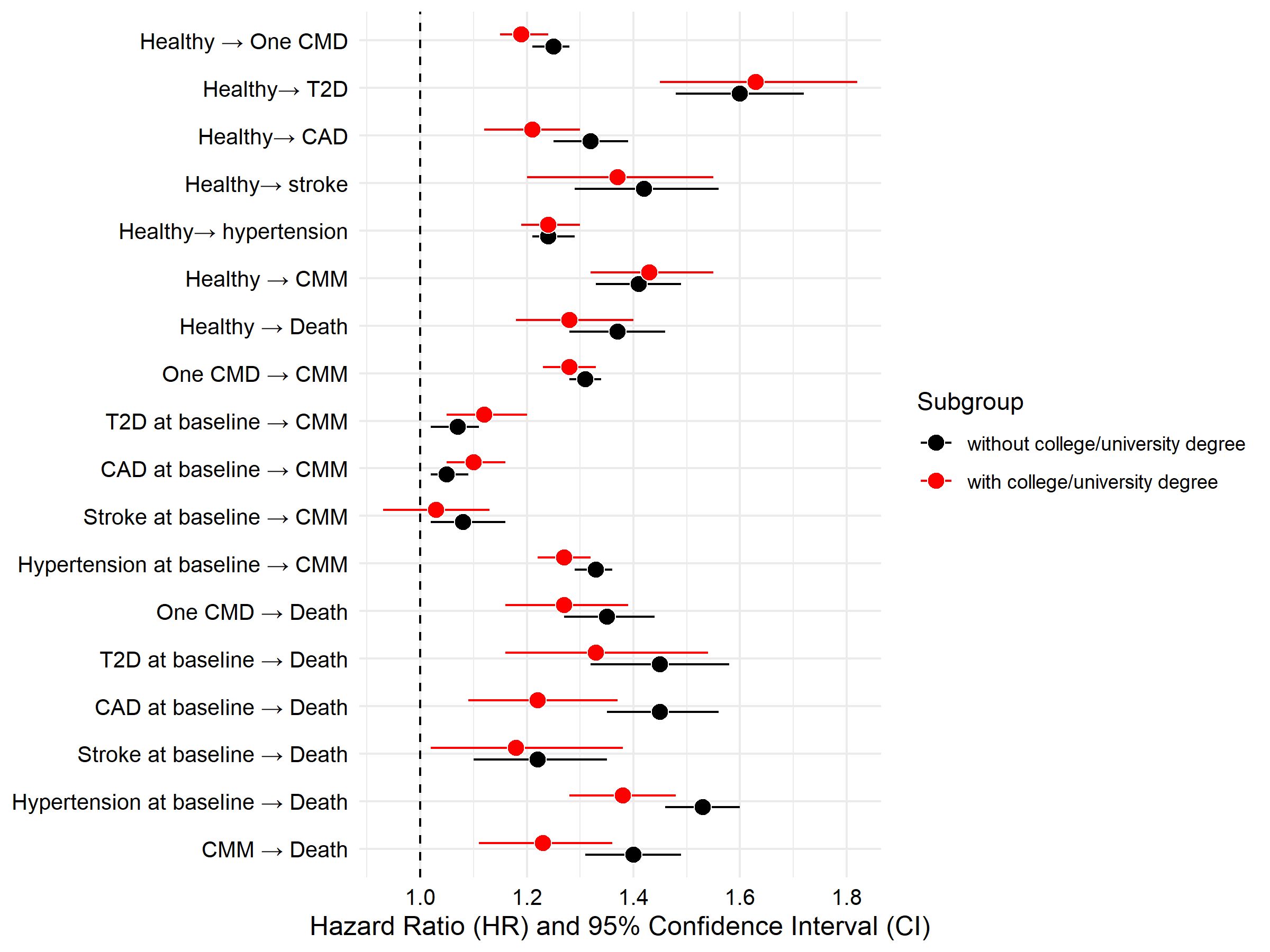


Figure S7. Subgroup analyses by education background for the association of Townsend deprivation index with one CMD, CMM and all-cause mortality from different baseline conditions. Note: CMD: cardiometabolic disease; CMM, cardiometabolic multi-morbidity. The HR values correspond to the hazard ratios of the fourth quartile (Q4) compared to the first quartile (Q1).


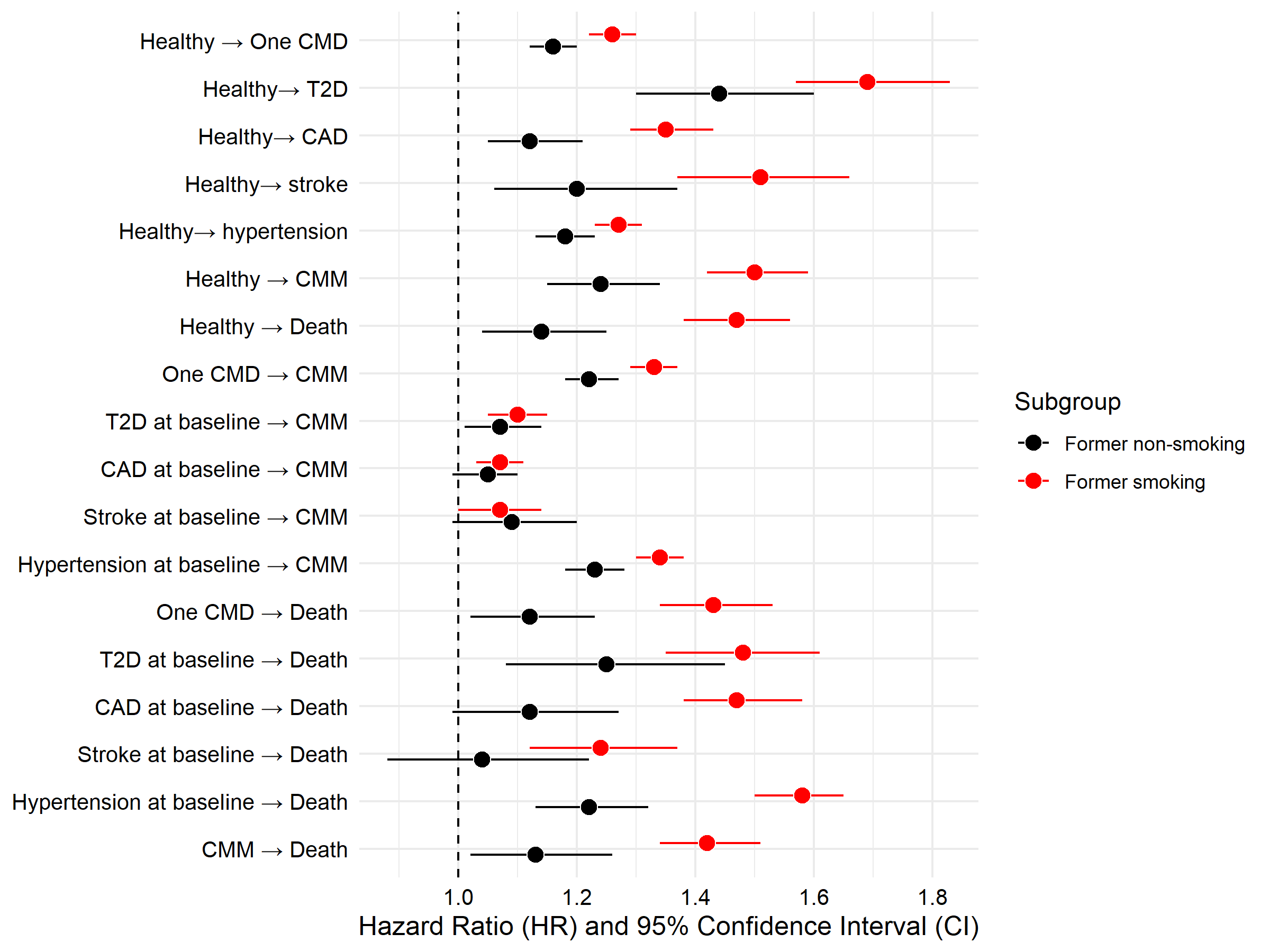


Figure S8. Subgroup analyses by smoking status for the association of Townsend deprivation index with one CMD, CMM and all-cause mortality from different baseline conditions. Note: CMD: cardiometabolic disease; CMM, cardiometabolic multi-morbidity. The HR values correspond to the hazard ratios of the fourth quartile (Q4) compared to the first quartile (Q1).


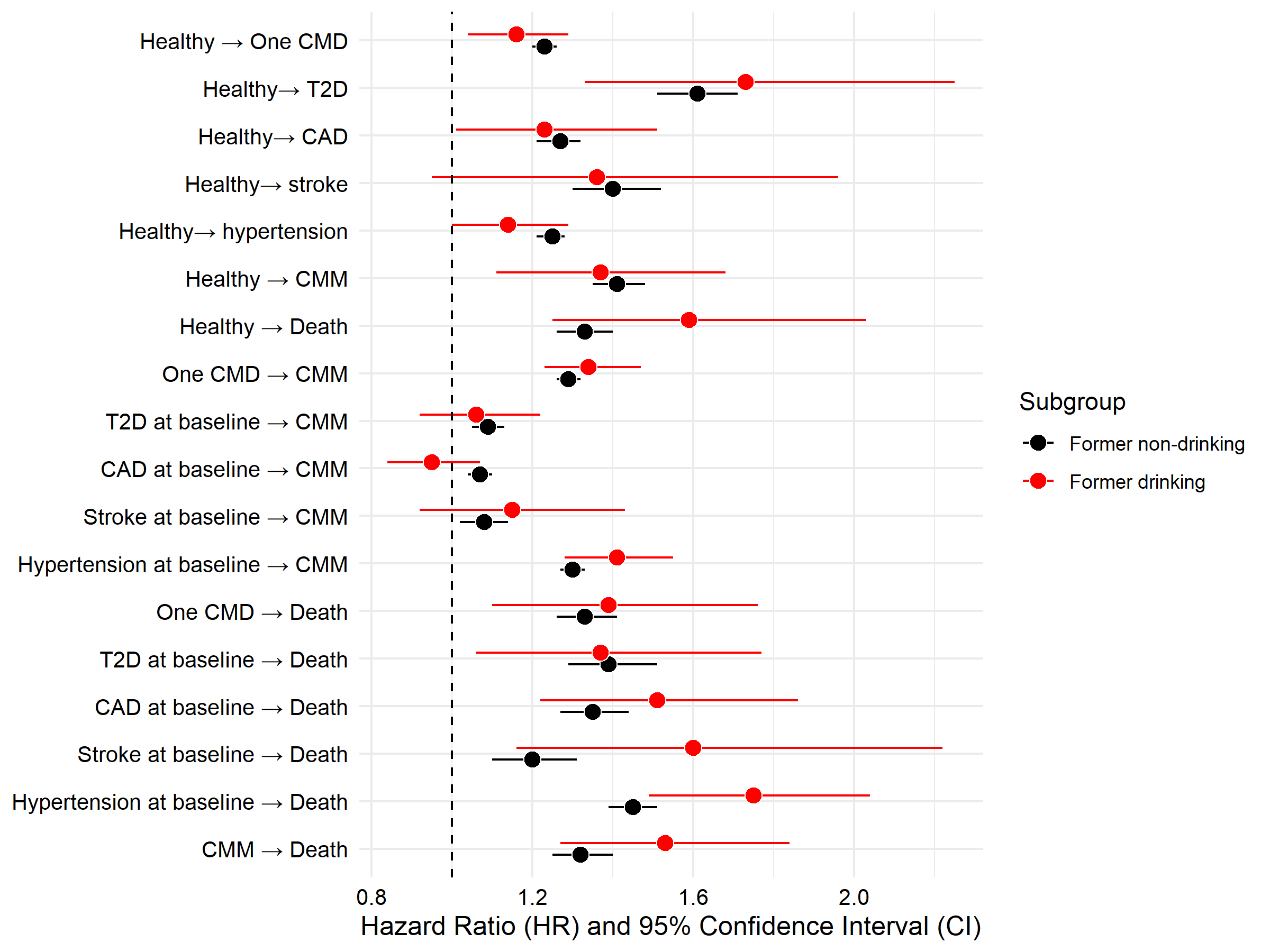


Figure S9. Subgroup analyses by drinking status for the association of Townsend deprivation index with one CMD, CMM and all-cause mortality from different baseline conditions. Note: CMD: cardiometabolic disease; CMM, cardiometabolic multi-morbidity. The HR values correspond to the hazard ratios of the fourth quartile (Q4) compared to the first quartile (Q1)


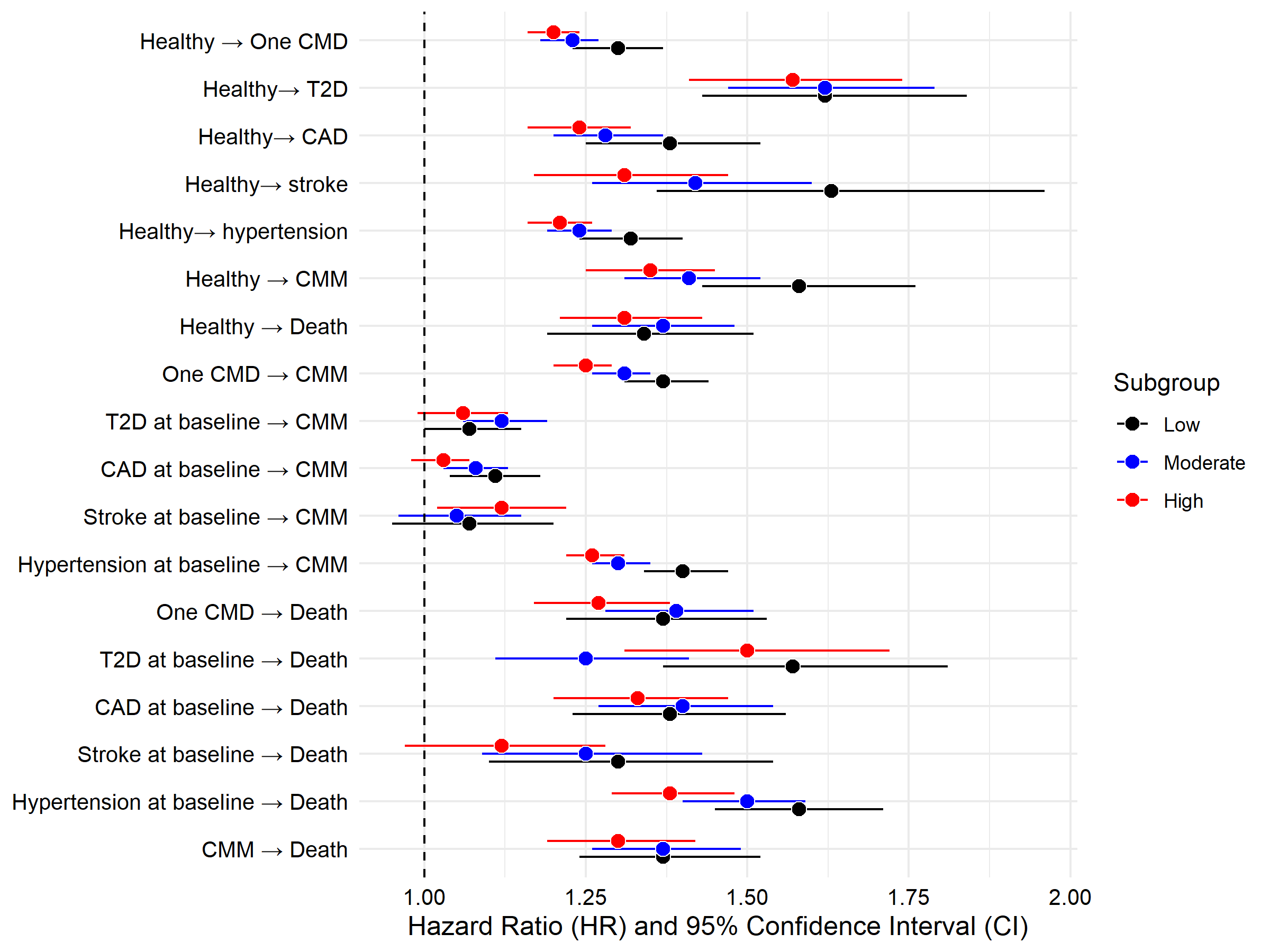


Figure S10. Subgroup analyses by physical activity for the association of Townsend deprivation index with one CMD, CMM and all-cause mortality from different baseline conditions. Note: CMD: cardiometabolic disease; CMM, cardiometabolic multi-morbidity. The HR values correspond to the hazard ratios of the fourth quartile (Q4) compared to the first quartile (Q1).


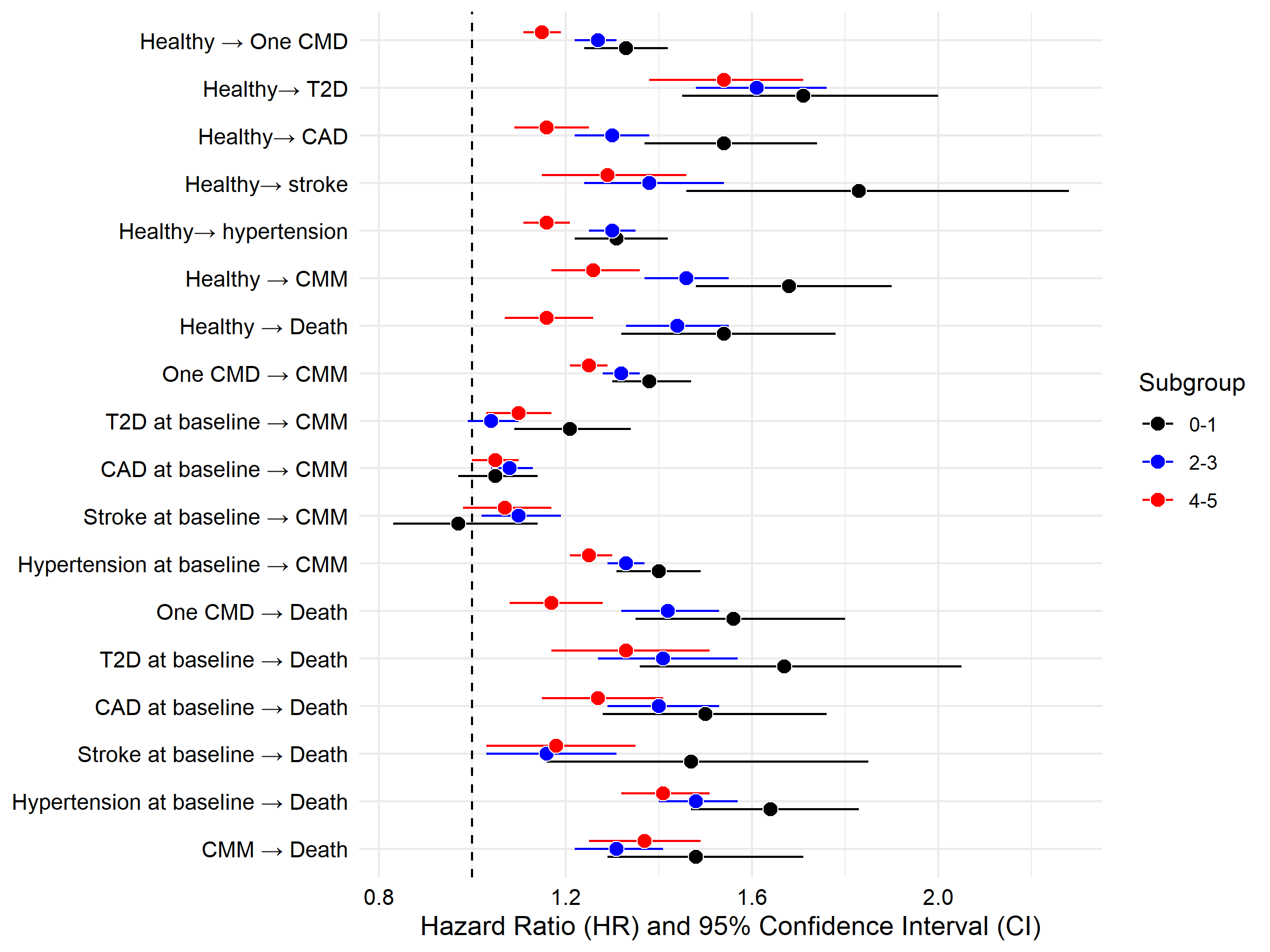


Figure S11. Subgroup analyses by healthy diet score for the association of Townsend deprivation index with one CMD, CMM and all-cause mortality from different baseline conditions. Note: CMD: cardiometabolic disease; CMM, cardiometabolic multi-morbidity. The HR values correspond to the hazard ratios of the fourth quartile (Q4) compared to the first quartile (Q1).


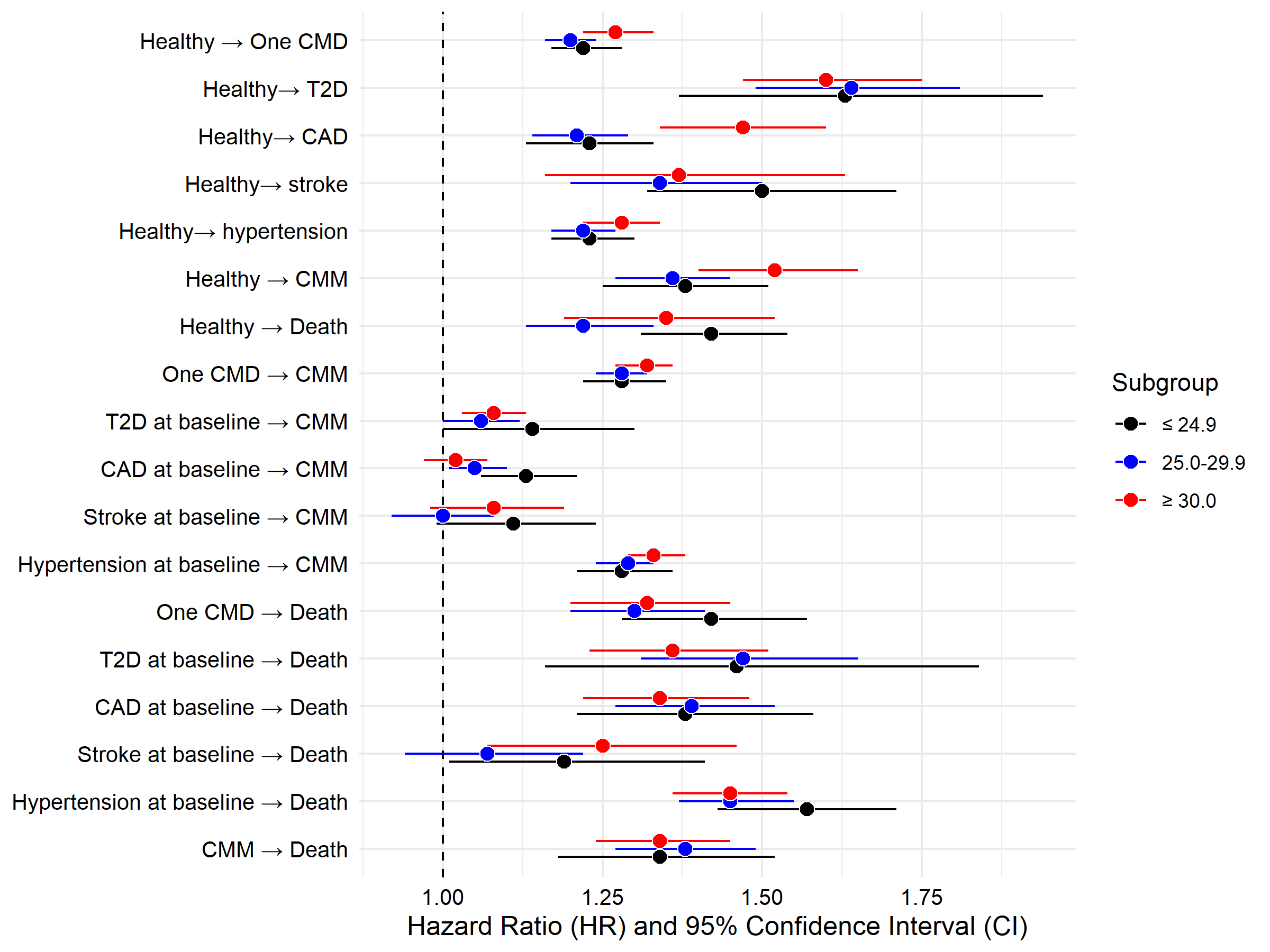


Figure S12. Subgroup analyses by BMI for the association of Townsend deprivation index with one CMD, CMM and all-cause mortality from different baseline conditions. Note: CMD: cardiometabolic disease; CMM, cardiometabolic multi-morbidity. The HR values correspond to the hazard ratios of the fourth quartile (Q4) compared to the first quartile (Q1).


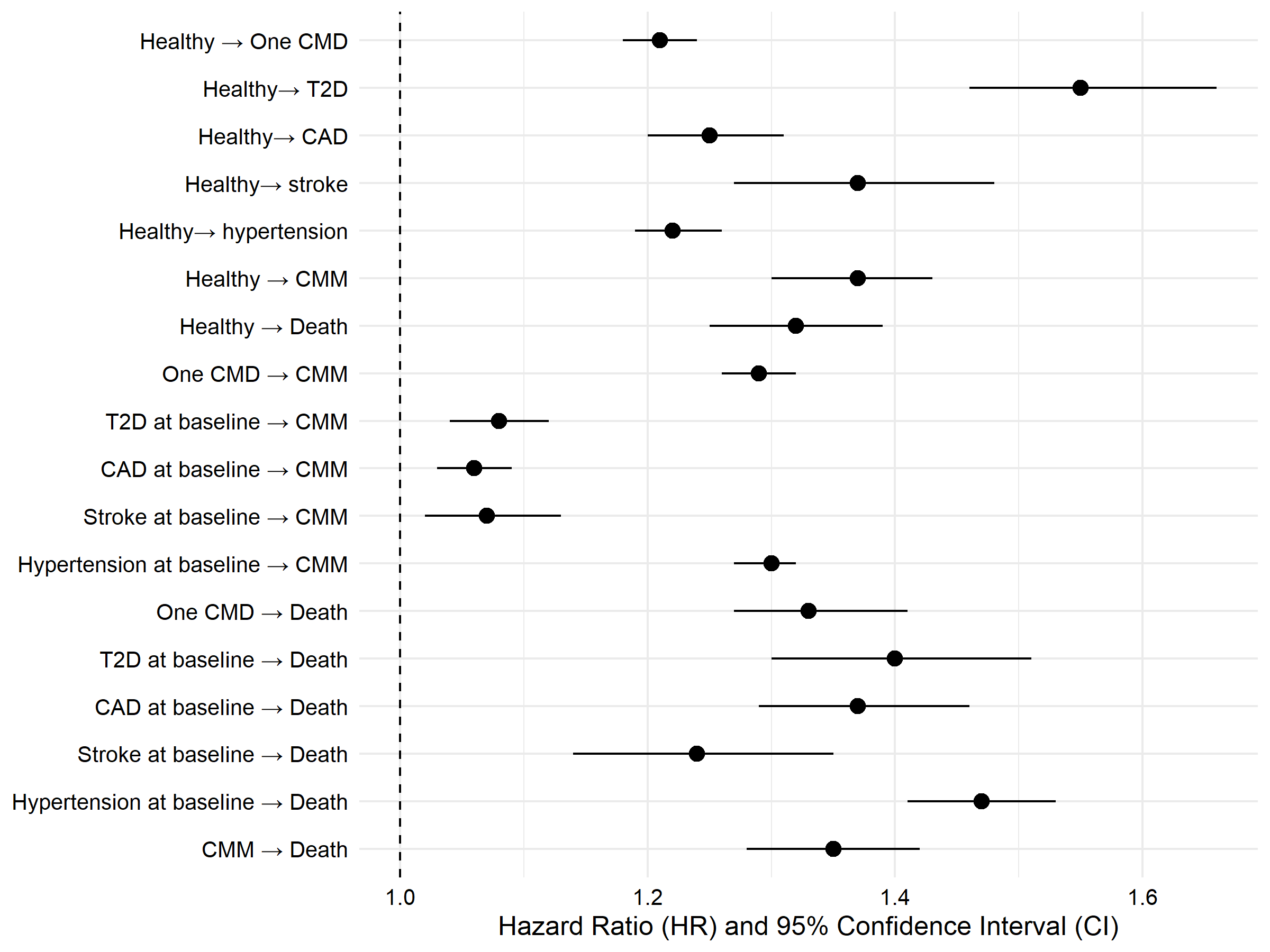


Figure S13. Analyses in white ethnicity for the association of Townsend deprivation index with one CMD, CMM and all-cause mortality from different baseline conditions. Note: CMD: cardiometabolic disease; CMM, cardiometabolic multi-morbidity. The HR values correspond to the hazard ratios of the fourth quartile (Q4) compared to the first quartile (Q1).


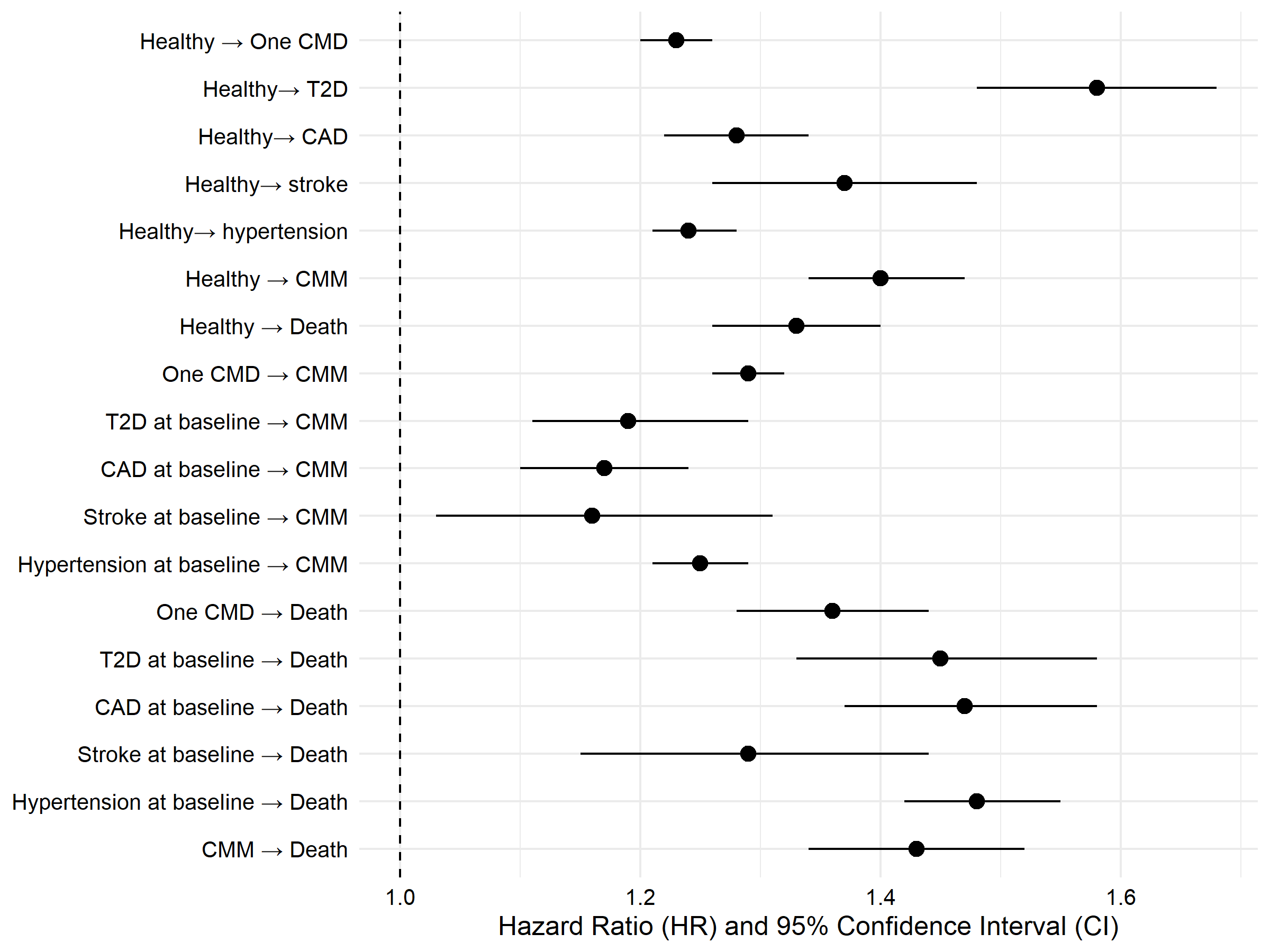


Figure S14. Sensitivity analyses for the association of Townsend deprivation index with one CMD, CMM and all-cause mortality from different baseline conditions while excluding events occurred within the first two years of follow-up. Note: CMD: cardiometabolic disease; CMM, cardiometabolic multi-morbidity. The HR values correspond to the hazard ratios of the fourth quartile (Q4) compared to the first quartile (Q1).


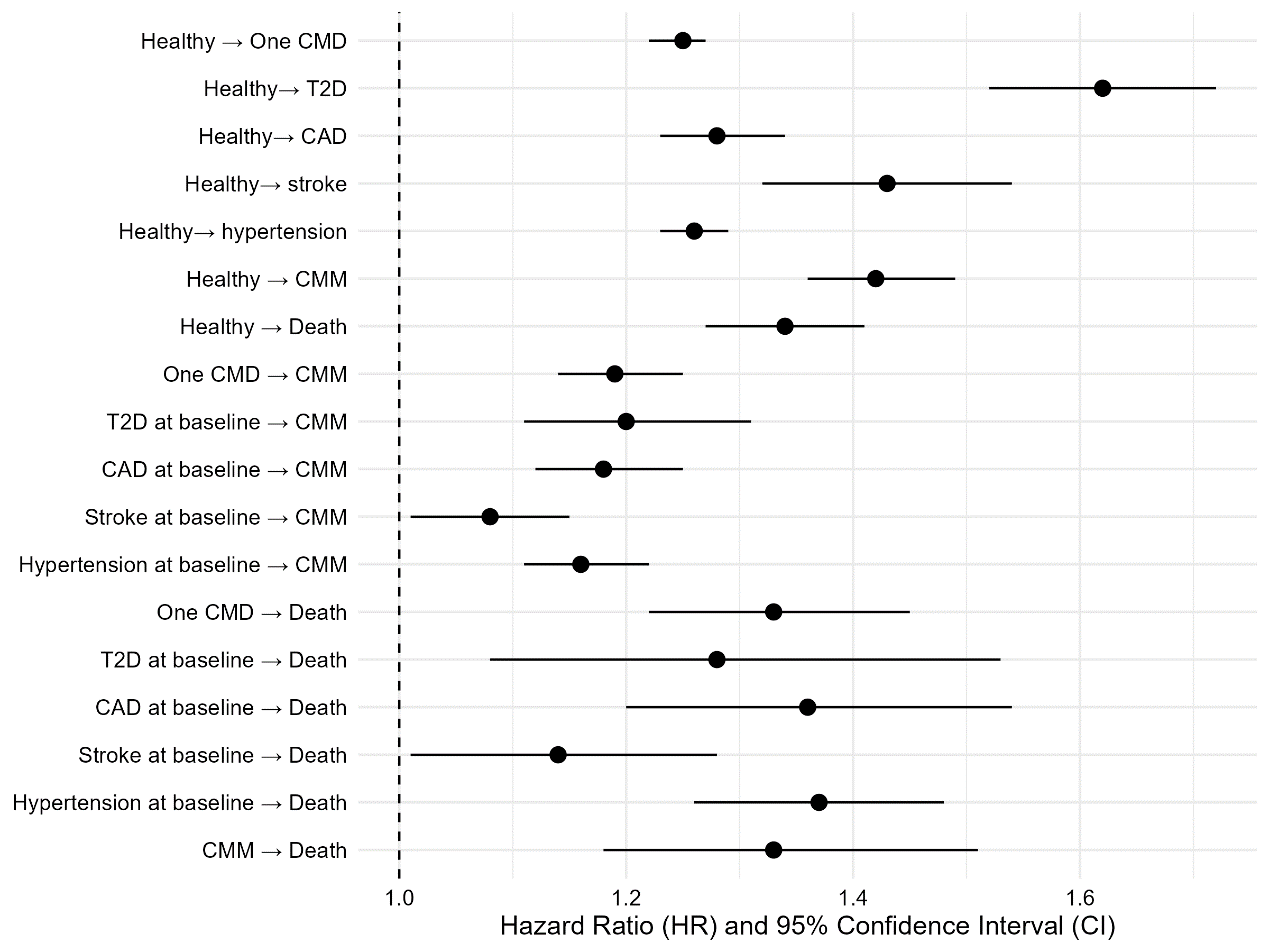


Figure S15. Sensitivity analyses for the association of Townsend deprivation index with one CMD, CMM and all-cause mortality from different baseline conditions only according to ICD-10 codes. Note: CMD: cardiometabolic disease; CMM, cardiometabolic multi-morbidity. The HR values correspond to the hazard ratios of the fourth quartile (Q4) compared to the first quartile (Q1).


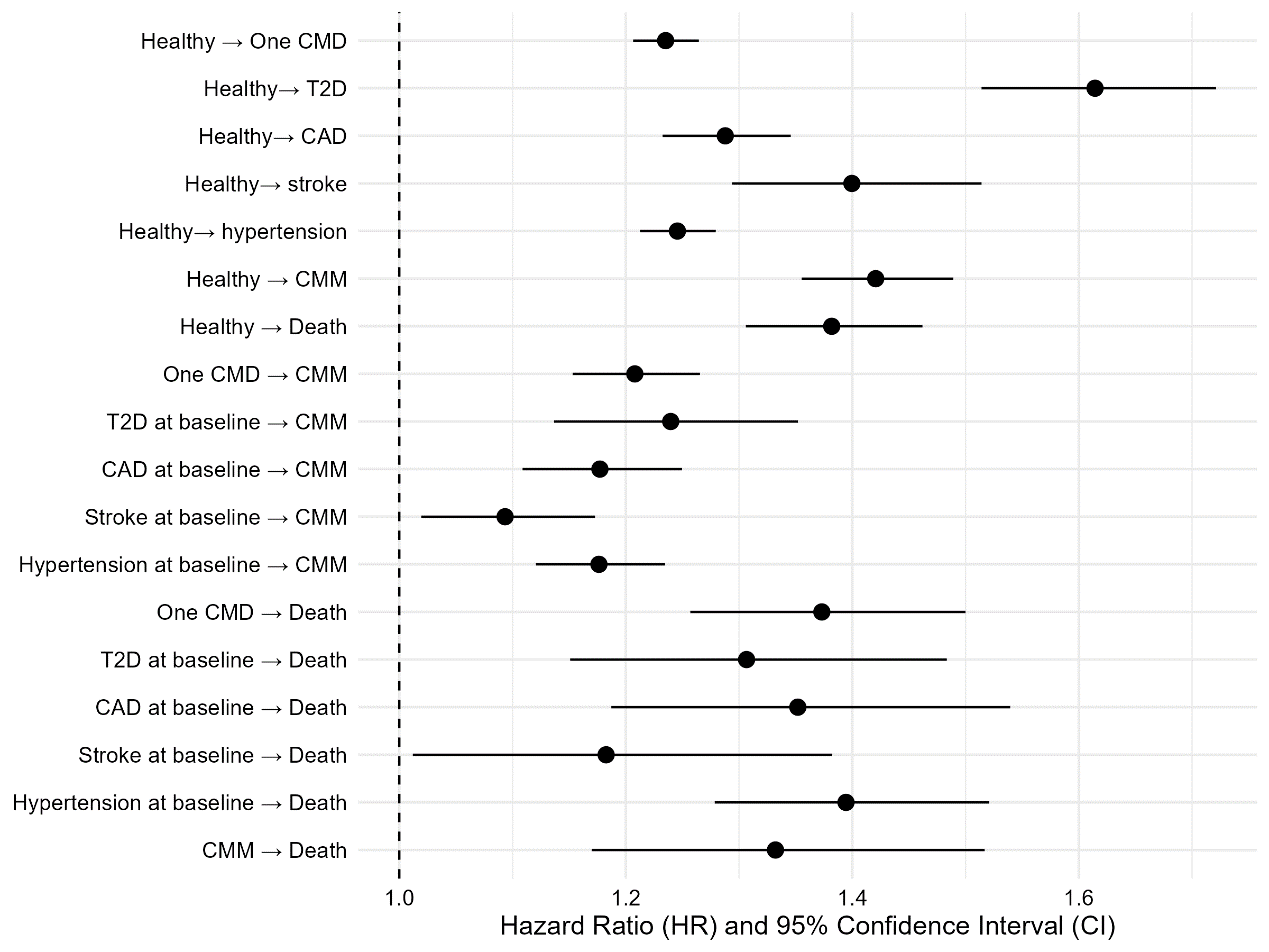


Figure S16. Sensitivity analyses for the association of Townsend deprivation index with one CMD, CMM and all-cause mortality from different baseline conditions while excluding individuals with cancer or dementia at recruitment. Note: CMD: cardiometabolic disease; CMM, cardiometabolic multi-morbidity. The HR values correspond to the hazard ratios of the fourth quartile (Q4) compared to the first quartile (Q1).

**References**

1. **IPAQ scoring protocol - International Physical Activity Questionnaire** [<https://sites.google.com/site/theipaq/scoring-protocol>]

2. Wang M, Zhou T, Song Y, Li X, Ma H, Hu Y, Heianza Y, Qi L: **Joint exposure to various ambient air pollutants and incident heart failure: a prospective analysis in UK Biobank**. *Eur Heart J* 2021, **42**(16):1582-1591.

3. Warrington NM, Beaumont RN, Horikoshi M, Day FR, Helgeland Ø, Laurin C, Bacelis J, Peng S, Hao K, Feenstra B *et al*: **Maternal and fetal genetic effects on birth weight and their relevance to cardio-metabolic risk factors**. *Nat Genet* 2019, **51**(5):804-814.
